# A Casson nanofluid flow within the conical gap between rotating surfaces of a cone and a horizontal disc

**DOI:** 10.1038/s41598-022-15094-w

**Published:** 2022-07-04

**Authors:** Galal M. Moatimid, Mona A. A. Mohamed, Khaled Elagamy

**Affiliations:** Department of Mathematics, Faculty of Education, Ain Shams University, Roxy, Cairo, Egypt

**Keywords:** Materials science, Mathematics and computing, Nanoscience and technology, Physics

## Abstract

The present study highlights the flow of an incompressible nanofluid following the non-Newtonian flow. The non-Newtonian fluid behavior is characterized by the Casson prototype. The flow occupies the conical gap between the rotating/stationary surfaces of the cone and the horizontal disc. Heat and mass transfer is also considered. The novelty of the proposed mathematical model is supplemented with the impacts of a uniform magnetic field imposed vertically upon the flow together with Ohmic dissipation and chemical reactions. The constitutive equations of the Casson fluid have been interpreted along with the cylindrical coordinates. The governing partial differential equations of momentum, energy, and concentration are converted into a set of nonlinear ordinary differential equations via appropriate similarity transformations. This scheme leads to a set of coupled nonlinear ordinary equations concerning velocity, temperature, and nanoparticles concentration distributions. These equations are analytically solved by means of the Homotopy perturbation method (HPM). The theoretical findings are presented in both graphical and tabular forms. The main objective of this study is to discuss the effects of the rotations of both cone and disc and the effects of the other parameters in the two cases of rotation alternatively. Additionally, the effect of the angle between the cone and the disk is one of our interesting points because of the importance of its effect in some engineering industry applications. The rotation parameters are found to have reduction effects on both the temperature and the radial velocity of the fluid, while they have an enhancing effect on the azimuthal velocity. The effects of other parameters with these rotations are found to be qualitatively the same as some earlier published studies. To validate the current mathematical model, a comparison with the previous scientific reports is made.

## Introduction

During the last decades, a lot of research has been made on the nanofluid flow and heat transfer with water as a base fluid. The term “nanofluids” was coined in 1995 by Choi and Eastman^1^. They were the first to use nano-sized small particles of metals, carbides, and oxides in the base fluids to enrich the thermal conductivity. Yu et al.^2^ investigated several metal and metal oxide nanoparticles in numerous base fluids and found satisfactory results, but many questions concerning these nano-structured material suspensions are still unexplored. Their work aimed to enhance the thermophysical properties such as thermal conductivity, thermal diffusivity, viscosity, and convective heat transfer coefficients when compared to base fluids such as oil, water or and ethylene glycol. The majority of nanofluid models only incorporate one or two proposed mechanisms of heat transfer. There has not been much basic research on determining the effective thermal diffusivity of nanofluids or heat transfer coefficients of nanofluids in natural convection; for instance, see Wong et al.^3^. Donzelli et al.^4^ revealed that a specific class of nanoparticles can be employed as a smart material to control the flow of heat by acting as a heat valve. Many publications on nanofluids focus on getting a better understanding of their behaviors, so they can be used in several areas such as nuclear reactors, transportation, electronics, biomedicine, and food. Wong and De Leon^5^ focused on presenting a wide range of current and prospective nanofluid uses, highlighting their better heat transfer potentials that are governable as well as the particulars that nanofluids possess, making them appropriate for such applications. Additionally, nanofluids as smart fluids, where heat transmission can be concentrated or enhanced, have also been reported. Ellahi et al.^6^ examined natural convection, where carbon nanotubes float in a water-based solution and flow through a vertical cone. Turkyilmazoglu^7^ explored the Buongiorno model of nanofluid with the influence of mass and heat transfer. Nazar et al.^8^ investigated a uniform Magnetohydrodynamic (MHD) flow with heat and mass transfer through a flowing narrow needle in a combination of nanomaterials driven by thermal radiation and viscous dissipation. Bilal et al.^9^ investigated the non-Newtonian Williamson nanofluid flow over an expanding sheet using an electro-magnetohydrodynamic flow. Because of the increased temperature transfer, thermal and solutal stratification effects as well as the impact of varying viscosity and Ohmic dissipation were considered. Vo et al.^10^ used a permeable region to represent a nanomaterial with varied forms. A magnetic force was applied to a nanofluid inside a porous gap. Dinarvand^11^ investigated a new type of nanofluid known as hybrid nanofluids, which are made by suspending two or more types of nanoparticles and hybrid nanoparticles in the considered base fluid. The current work is expected to get an improved physical understanding of the flow process, which will support the design of flow and heat transfer technology.

The well-known MHD system is mostly employed in the Fluid Mechanics models that represent the behavior of electrically conducting fluids in the presence of a magnetic field. The communication of electrically conducting fluids and electromagnetic fluids is the subject of MHD. When a conducting fluid passes through a magnetic field, an electric field is created and results in the current, which joins with the magnetic field to produce a body force. Because of the physical significance of MHD, complexity, and mathematical challenges, many efforts have been made in this discipline. After the incorporation of nanofluids, which extended the study of this topic, this significance increased dramatically. Amine et al.^12^ investigated the behavior of a triangle cavity containing an Ag-MgO/water hybrid nanofluid with a revolving circular boundary under MHD natural convection, with a right-angled corner containing a quarter circular porous medium and kept at a uniform hot temperature. Along with the vast variety of MHD applications in astrophysics, plasma, and other fields, physicists and mathematicians have conducted several studies. Numerous mathematical conclusions concerning the compressible MHD system have been published so far; see Chen and Wang^13^ and the sources referenced herein. Electromagnetic pumping, metallurgy, nuclear fusion reactors, and power generation are just a few examples of the typical industrial applications of MHD^14,15^. Aluminum reduction cells and electromagnetic launching are two further applications. The widespread usage of MHD is due to the fact that it is a non-invasive method of controlling the flow of conducting fluids. Nadeem and Saleem^16^ demonstrated an MHD nanofluid flow over a rotating cone with the thermophoresis and Brownian interactions. Towers and Garrett^17^ investigated similarity solutions of compressible laminar flows subject to surface mass flux over a collection of rotating cones. Astrophysics, controlled thermonuclear reactions, and industry are three principal applications of MHD^18^. Plasma and magnetic fields exist in the universe, stars, and interstellar gas. Accordingly, MHD was created and employed first in the fields of astrophysics, solar physics, and geophysics. Ramzan et al.^19^ discussed the flow of the nano ferrofluid flow in various geometries. This channel was narrowed down if the flow of the nano ferrofluid flows over a stretching rotating disk with the Hall current and low oscillating magnetic field. In the effect of heat radiation, Yazdi et al.^20^ investigated two-dimensional convection flow MHD boundary layer with a stagnation-point flow across a stretched vertical plate in a porous medium filled with a nanofluid. The fundamental equations of motion were reduced to a set of nonlinear ordinary differential equations using a similarity transform. In accordance with the diverse applications of MHD in numerous practical applications, the current work is conducted in the presence of the MHD.

Casson fluid is a non-Newtonian fluid, where the shear stresses are in a nonlinear relation to the velocity gradients. The investigation of Casson fluid has a wide range of engineering applications. Casson fluid is among the most significant non-Newtonian fluids, and has a wide range of applications in biomechanics, plastic, and metals. It has evoked the interest of many academics because of its uses in food preparation, metallurgy, drilling, and biotechnology^21,22^. The Casson fluid prototype is the greatest precise mathematical communication for examining the dynamics of fluids with non-zero plastic dynamic viscosity, which is much closer to blood. Additionally, it contains plasma and protein and can be used to make coals in water, paints, synthetic lubricants, and biological fluids, including tomato sauce, honey, soup, jelly, and blood. The connection between stress and the rate of strain is a nonlinear Casson constitutive equation, which was derived by Casson^23^. Tao^24^ investigated the difficulties of mixed free and forced convection in channels by developing a complex function that is clearly relevant to velocity, temperature fields, and the Helmholtz wave equation, which combines momentum and the energy equation in the complex domain. Walawender et al.^25^ examined an approximate Casson fluid model of blood tube, in which the pressure drops, and volumetric flow rate were determined experimentally. Batra and Jena^26^ evaluated a steady laminar flow of a Casson fluid in a slightly rounded tube of a circular cross section, which was analyzed for a large Dean number. The governing equations of motion were analyzed by a methodology of a numerical technique. On the motion of Casson fluid over a rotating non-uniform surface, Oke et al.^27^ reported the importance of raising Coriolis force and reducing plastic dynamic viscosity as well as the Prandtl number and buoyancy forces. Additionally, the second law of MHD Casson flow above a stretching sheet was studied through velocity slip^28^. Their findings revealed that the Casson parameter progresses heat transfer. The effects of the MHD flow and ion slip on a 3D Casson nanofluid flow across a linear extended surface with a modified Fourier law and surface stimulated chemical reaction in a porous layer were investigated by Ramzan et al.^29^. The magnetized and non-magnetized Casson fluid flow with gyrotactic microorganisms over a layered stretched cylinder was presented by Dawar et al.^30^. On a two-dimensional electrically conducting radiative Casson nano liquid flow across a flexible cylinder encased in a porous media, Shaheen et al.^31^ investigated the impacts of varied features combined with chemical reaction and Kinetics activation energies. The current study is investigated in the context of the Casson fluid, in conformity with the widespread applications of MHD in different real-world applications.

Because most nonlinear differential equations do not have analytical solutions, approximation and numerical approaches are frequently used. Because many nonlinear equations do not have a small parameter, all classical perturbation techniques require it, which restricts the use of all traditional perturbation methods. The determination of a small parameter is a difficult operation that necessitates the employment of special techniques. Many studies^32,33^ have employed HPM to overcome these challenges. HPM provides a number of auxiliary parameters that can be used to control the convergence of solution series^34,35^. By integrating the traditional Homotopy and the perturbation approach, He^36–38^ established a modified HPM by addressing nonlinear initial and boundary-value problems. In most instances, a quick convergent series solution may be achieved by using this approach. For numerical computation, only a few terms of the series are usually employed. A Duffing equation was utilized to demonstrate the effectiveness and convenience. The results showed that the suggested method, in the first-order approximation, is universally valid even for very large parameters and is much more efficient than the ordinary perturbation solution. Only one iteration was required to attain the high accuracy of the solutions. A basic Homotopy was generated using the modified Lindstedt-Poincaré method technique. The linear solution terms and coefficients were expanded into a series of embedding parameters. The two-point boundary-value problems appear in a wide range of engineering and practical physics. Consequently, Chun and Sakthivel^39^ used the HPM to solve two-point boundary-value problems that were linear and nonlinear. Their study compare**d** the performance of HPM with that of the extended Adomian decomposition method. Biazar and Ghazvini^40^ examined the convergence of the HPM. There were some available examples such as Berger, Schrödinger, and fourth-order parabolic partial differential equations. They validated the convergence hypothesis and demonstrated the simplicity and efficiency of the method. Moatimid^41^ examined the behavior of a sliding bead on a smooth vertical parabola. By connecting the HPM and Laplace transforms, with the aid of the nonlinear expanded frequency, a bounded analytic solution was attained. A modified HPM was utilized by Moatimid^42^ to get a better approximate solution of the Duffing oscillator. Additionally, he obtained the exact solution of the cubic Duffing equation. Due to the exceedingly difficult fundamental equations, the HPM is used to study the subject at hand for the sake of simplicity.

The flow of a nanofluid flow within the conical gap between the cone and the surface of a rotating disc has a range of useful and technical applications, including medical purposes^43^, the calculation of viscosity of fluid, the stability analysis of an Oldroyd-B creeping flow, and gas turbines in a conical diffuser in the cooling system to compress air. Therefore, through all the above-cited investigations and their applications, the aim of the present study is to find an analytical solution for a mixed convective flow in the presence of Casson fluid under the influence of the magnetic field and amplification using the HPM.

The current research focuses on Casson fluid flow incorporating nanoparticles over a gap between a revolving cone and disc in the realm of non-Newtonian fluids. This model is useful in some situations, such as measuring the viscosity or rheology of fluids with a cone-plate viscometer or rheometer. Ohmic dissipation, heat generation, magnetic field, and chemical reaction are also considered. At the disc, velocity slip circumstances are considered. In addition to our new findings, the conclusions include a comparison of our study with previously published research.

At the end of this study, the following questions are expected to be answered:

- How do the velocity components behave for a non-Newtonian (Casson) nanofluid in the gap between a rotating cone and disc?
- How are the temperature and nanoparticles distributed through the current flow?
- What are the impacts of the cone rotation and disc rotation on the velocity, temperature, and nanoparticles distributions?
- What are the impacts of the involved parameters on the above distributions in cases of a rotating cone/stationary disc and inversely with a rotating disc/stationary cone?

To crystallize the paper presentation, the rest of it is organized as follows: The problem methodology is explained in Sect. 2. This Section includes the controlling equations of motion as well as the reasonable boundary conditions. The boundary-value problem is discussed in Sect. 3 using appropriate transformations. Section 4 illustrates how to create an analytic solution using HPM. The findings and discussions are presented in Sect. 5. Finally, the major findings are summarized as concluding observations in Sect. 6.

## Mathematical representation of the model

**Figure 1:**
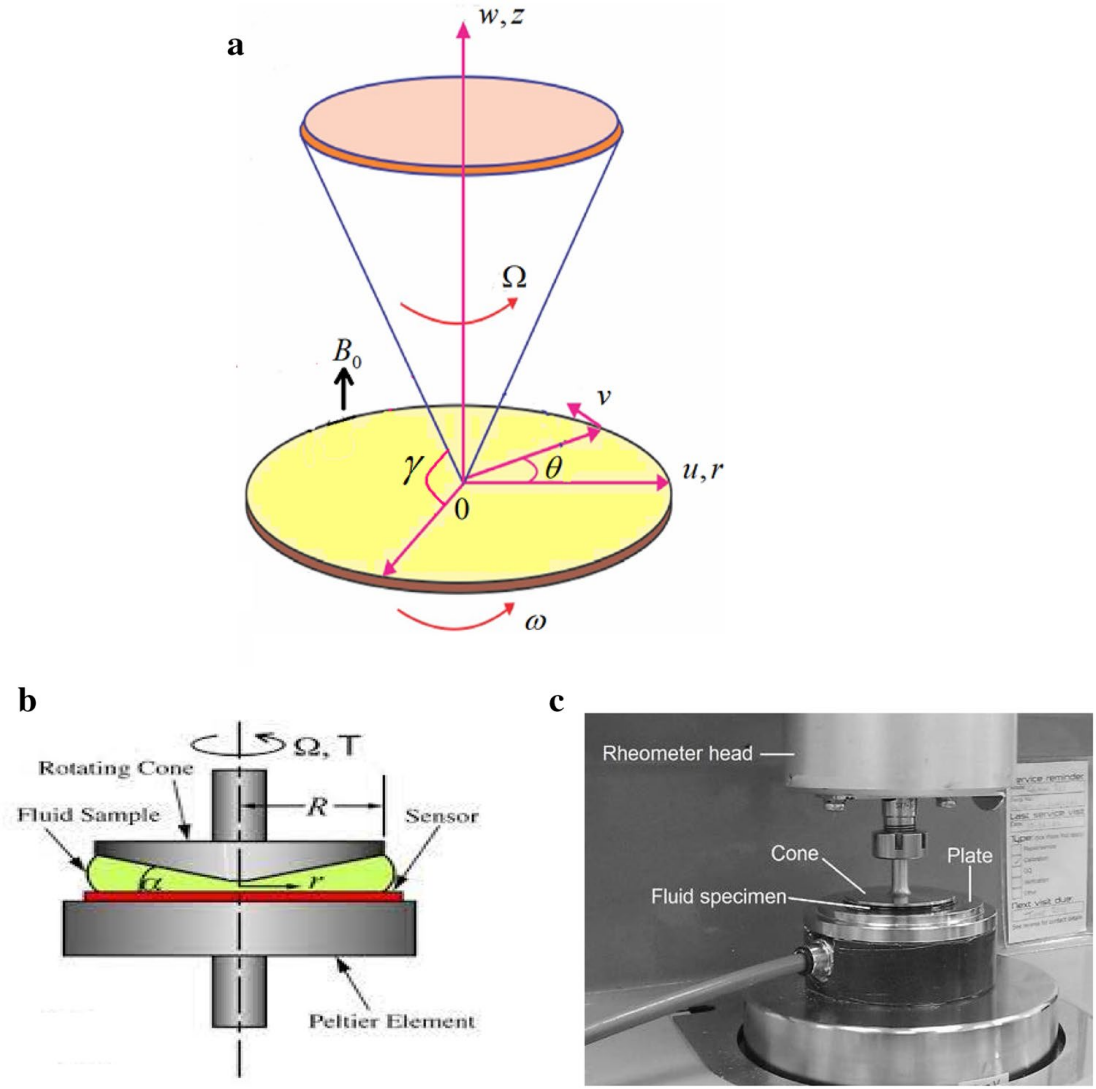
(**a**) Physical flow configuration. (**b**) Graphical model of a cone-plate rheometer^44,45^. (**c**) Actual model of a cone-plate rheometer^44,45^.

A steady, non-Newtonian hydrodynamic flow in the gap between a cone and a disc conforming Casson nanofluid is considered. For more convenience, we work with cylindrical coordinates. For the sake of simplicity, the full axi-symmetric equations of fluid flow configuration are reflected. The disc is positioned at $z = 0$ and rotates with a uniform angular velocity $\omega$ around its normal direction, while it moves in the radial direction with a constant velocity $U_{w}$. At the same time, the cone rotates with another uniform angular velocity $\Omega$ around its axial direction. Along with this construction, for the sake of simplicity, the pressure gradient may be neglected. Additionally, heat transfer is reviewed with the addition of Ohmic dissipation, while the nanoparticles volume fraction is computed due to the chemical reactions. The rotating disc is kept at constant temperature and constant concentration, with the cone wall being at different constant temperature and constant concentration. Additionally, a magnetic field of uniform strength $B_{0}$ is designed. For the sake of straightforwardness, the electric field may be neglected. The absence of the induced magnetic force is caused due to the assumption of a smaller Reynolds number. The aim of this study is to understand and solve the problem of thermal and volumetric nanoparticles flow of the Casson fluid between the two rotating surfaces, i.e., disc and cone which are assumed to rotate with a gap angle $\gamma$ between them. The physical situation of the flow model is clarified and shown in Fig. 1a.

### Governing equations of motion and appropriate boundary conditions

The rheological equation of state of Casson fluid can be defined by Casson^23^ as follows:

1 $$\tau_{ij} = \left\{ {\begin{array}{*{20}c} {2\left( {\mu_{0} + \frac{{P_{y} }}{{\sqrt {2\pi } }}} \right)e_{ij} \,\,\,\,\,\,{\text{when}}\,\,\,\,\,\pi > \pi_{c} ,} \\ {2\left( {\mu_{0} + \frac{{P_{y} }}{{\sqrt {2\pi_{c} } }}} \right)e_{ij} \,\,\,\,\,{\text{when}}\,\,\,\,\,\,\pi < \pi_{c} ,} \\ \end{array} } \right.$$

where $P_{y}$ is known as yield stress of the fluid and is expressed mathematically by Fung^46^ as:

2 $$P_{y} = \frac{{\mu_{0} \sqrt {2\pi_{c} } }}{\beta }$$

where $e_{ij}$ s the deformation rate tensor, $\pi$ is the product of the component of deformation rate by itself (i.e. $\pi = e_{ij} e_{ij}$), $\pi_{c}$ is the critical value based on the non-Newtonian model and $\beta$ is the casson parameter.

Casson fluid can also be made of human blood. Human red blood cells can relax as a result of the presence of any chemicals in fluid base plasma, such as protein, fibrinogen, and globulin. If the intercellular acts like a plastic solid, a yield stress exists that may be detected as a Casson fluid continuous yield stress, see Pramanik^47^. As mentioned above, as shown by Animasaun^48^, the blood viscosity may be expressed as:

3 $$\mu = \mu_{0} + \frac{{P_{y} }}{{\sqrt {2\pi_{c} } }}\,\,\,\,{\text{when}}\,\,\,\pi < \pi_{c}$$

Buongiorno^49^ investigated the convective transport of nanofluids considering seven different slip mechanisms such as inertia, Brownian diffusion, thermophoresis, diffusiophoresis, Magnus effect, fluid drainage, and gravity. Among these mechanisms, only Brownian diffusion and thermophoresis were found to be significant. Furthermore, the study showed that energy transfer by nanoparticles dispersion is negligible and cannot explain the abnormal heat transfer coefficient.

In accordance with the abovementioned relations, the governing equations of motion**,** the conservation of mass, the momentum of an incompressible non–Newtonian fluid, the energy equation with the Joule heat and heat source/sink effects and the nanoparticles volume fraction equation with Brownian and thermophoresis and chemical reaction effects may be considered as follows:

The incompressibility equation as El-Dabe et al.^50^ produces is

4 $$\frac{\partial u}{{\partial r}} + \frac{u}{r} + \frac{\partial w}{{\partial z}} = 0$$

The momentum equations as El-Dabe et al.^50^ in the radial direction provides are

5 $$\rho \left( {u\frac{\partial u}{{\partial r}} + w\frac{\partial u}{{\partial z}} - \frac{{v^{2} }}{r}} \right) = \mu_{0} \left( {1 + \beta^{ - 1} } \right)\left( {\frac{{\partial^{2} u}}{{\partial r^{2} }} + \frac{{\partial^{2} u}}{{\partial z^{2} }} + \frac{1}{r}\frac{\partial u}{{\partial r}} - \frac{u}{{r^{2} }}} \right) - \sigma B_{o}^{2} u$$

the azimuthal direction gives

6 $$\rho \left( {u\frac{\partial v}{{\partial r}} + w\frac{\partial v}{{\partial z}} + \frac{uv}{r}} \right) = \mu_{0} \left( {1 + \beta^{ - 1} } \right)\left( {\frac{{\partial^{2} v}}{{\partial r^{2} }} + \frac{{\partial^{2} v}}{{\partial z^{2} }} + \frac{1}{r}\frac{\partial v}{{\partial r}} - \frac{v}{{r^{2} }}} \right) - \sigma B_{o}^{2} v$$

and the axial direction yields

7 $$\rho \left( {u\frac{\partial w}{{\partial r}} + w\frac{\partial w}{{\partial z}}} \right) = \mu_{0} \left( {1 + \beta^{ - 1} } \right)\left( {\frac{{\partial^{2} w}}{{\partial r^{2} }} + \frac{{\partial^{2} w}}{{\partial z^{2} }} + \frac{1}{r}\frac{\partial w}{{\partial r}}} \right)$$

The energy equation by Refs^50–52^. is as follows:

8 $$\begin{gathered} \,\,\,\,\,\,\,\,\,\,u\frac{\partial T}{{\partial r}} + w\frac{\partial T}{{\partial z}} = \frac{\alpha }{{(\rho c)_{f} }}\left( {\frac{{\partial^{2} T}}{{\partial r^{2} }} + \frac{1}{r}\frac{\partial T}{{\partial r}} + \frac{{\partial^{2} T}}{{\partial z^{2} }}} \right) + \hfill \\ \frac{{(\rho c)_{p} }}{{(\rho c)_{f} }}\left( {D_{B} \left( {\frac{\partial T}{{\partial r}}\frac{\partial C}{{\partial r}} + \frac{\partial T}{{\partial z}}\frac{\partial C}{{\partial z}}} \right) + \frac{{D_{T} }}{{T_{\infty } }}\left( {\left( {\frac{\partial T}{{\partial r}}} \right)^{2} + \left( {\frac{\partial T}{{\partial z}}} \right)^{2} } \right)} \right) + \hfill \\ \,\,\,\,\,\,\,\,\,\,\,\,\,\,\,\,\,\,\,\frac{{\sigma B_{0}^{2} }}{{(\rho c)_{f} }}\left( {u^{2} + v^{2} } \right) + \frac{{Q_{1} }}{{(\rho c)_{f} }}\left( {T - T_{\infty } } \right) \hfill \\ \end{gathered}$$

Additionally, the nanoparticles concentration equation by Refs^50–52^. gives:

9 $$u\frac{\partial C}{{\partial r}} + w\frac{\partial C}{{\partial z}} = \,D_{B} \left( {\frac{{\partial^{2} C}}{{\partial r^{2} }} + \frac{1}{r}\frac{\partial C}{{\partial r}} + \frac{{\partial^{2} C}}{{\partial z^{2} }}} \right) + \frac{{D_{T} }}{{T_{\infty } }}\left( {\frac{{\partial^{2} T}}{{\partial r^{2} }} + \frac{1}{r}\frac{\partial T}{{\partial r}} + \frac{{\partial^{2} T}}{{\partial z^{2} }}} \right) - R_{1} (C - C_{\infty } )$$

### Physical quantities of interest

The physical quantities of interest in this investigation are the skin friction coefficients**,** respectively $Cf_{r}$ and $Cf_{\theta }$ along the $r$ and $\,\theta$ directions, the Nusselt number $Nu$ and the Sherwood number $Sh$, which are defined by Lv et al.^53^ as :

10 $$Cf_{r} = \tau_{r} \left| {_{z = 0} } \right./\rho_{f} (r\omega )^{2}$$

11 $$Cf_{\theta } = \tau_{\theta } \left| {_{z = 0} } \right./\rho_{f} (r\omega )^{2} ,$$

12 $$Nu = r\left. {\frac{\partial T}{{\partial z}}} \right|_{z = 0} /(T_{w} - T_{\infty } )$$

13 $$Sh = r\left. {\frac{\partial C}{{\partial z}}} \right|_{z = 0} /(C_{w} - C_{\infty } )$$

The preceding equations control the motion of a Casson nanofluid, and they should satisfy a set of boundary conditions. Gul et al.^54^ and Zainal et al.^55^ established the necessary boundary conditions. These conditions may be formulated as follows:

14 $$\left. {\begin{array}{*{20}c} {u = U_{w} ,v = r\omega ,w = 0,T = T_{w} ,C = C_{w} \,\,\,\,\,\,\,at\,\,\,z = 0} \\ {\,\,\,\,u = 0,v = r\Omega ,w = 0,\,\,T = T_{\infty } ,C = C_{\infty } \,\,\,\,\,at\,\,\,\,\,z = r\,{\text{tan}}\gamma } \\ \end{array} } \right\}$$

Now, our boundary-value problem becomes well-defined. The method of solution will be presented in the next section.

## Appropriate transformations of similarity

Through an adequately similarity transformation, the governing nonlinear partial differential equations turn into ordinary differential ones. The relevant similarity transformations can be created, based on the work of Gul et al.^54^ and Turkyilmazoglu^56^, as follows:

15 $$\left. \begin{gathered} u = \frac{\upsilon }{r}F(\eta ) = U_{w} F(\eta ),\,v = \frac{\upsilon }{r}G(\eta ),\,w = \frac{\upsilon }{r}H(\eta ), \hfill \\ \theta (\eta ) = \frac{{T - T_{\infty } }}{{T_{r} - T_{\infty } }},\,\varphi (\eta ) = \frac{{C - C_{\infty } }}{{C_{r} - C_{\infty } }},\,\,{\text{and}}\,\,\eta = \,\frac{z}{r}, \hfill \\ \end{gathered} \right\}$$

where $F,\,G,\,H$ are non-dimensional velocity components and $\eta$ is a non-dimensional similarity coordinate.

Inserting these transformations into the previous governing equations of motion, in order, one finds

16 $$H^{\prime} - \eta \,F^{\prime} = 0$$

17 $$(1 + \beta^{ - 1} )\left( {(1 + \eta^{2} )F^{\prime\prime} + 3\eta \,F^{\prime}} \right) + \eta \,F\,F^{\prime} + F^{2} - H\,F^{\prime} + G^{2} - M\,F = 0$$

18 $$(1 + \beta^{ - 1} )\left( {(1 + \eta^{2} )G^{\prime\prime} + 3\eta \,G^{\prime}} \right) + \eta \,F\,G^{\prime} - H\,G^{\prime} - M\,G = 0$$

19 $$(1 + \beta^{ - 1} )\left( {(1 + \eta^{2} )H^{\prime\prime} + 3\eta \,H^{\prime} + H} \right) + \eta \,F\,H^{\prime} - HH^{\prime} + H\,F = 0$$

20 $$\begin{gathered} (1 + \eta^{2} )\theta ^{\prime\prime} + \eta \,\theta ^{\prime} + \Pr (\eta \,F - H)\,\theta ^{\prime} + \Pr \,N_{B} \,(1 + \eta^{2} )\theta ^{\prime}\varphi ^{\prime} + \hfill \\ \Pr \,N_{T} \,(1 + \eta^{2} )\theta ^{{\prime}{2}} + \Pr \,Ec\,M(F^{2} + G^{2} ) + \Pr \,Q\,\theta = 0 \hfill \\ \end{gathered}$$

21 $$(1 + \eta^{2} )\varphi ^{\prime\prime} + \eta \varphi ^{\prime} + Sc(\eta F - H)\varphi ^{\prime} + \frac{{N_{T} }}{{N_{B} }}\left( {(1 + \eta^{2} )\theta ^{\prime\prime} + \eta \theta ^{\prime}} \right) - R_{c} Sc\,\varphi = 0$$

with the physical quantities of interest:

22 $$Cf_{r} = (1 + \beta^{ - 1} )F^{\prime}(0)/{\text{Re}}_{\omega }^{2}$$

23 $$Cf_{\theta } = (1 + \beta^{ - 1} )G^{\prime}(0)/{\text{Re}}_{\omega }^{2}$$

24 $$Nu = \theta ^{\prime}(0)$$

25 $$Sh = \varphi ^{\prime}(0)$$

where the dash denotes the differentiation with respect to the independent variable $\eta$.

Moreover, the boundary conditions as given in Eq. (14) have been converted as follows:

26 $$\left. {\begin{array}{*{20}c} {F(0) = 1,G(0) = {\text{Re}}_{\omega } ,H(0) = 0,\theta (0) = 1,\varphi (0) = 1} \\ {F(\eta_{0} ) = 0,G(\eta_{0} ) = {\text{Re}}_{\Omega } ,H(\eta_{0} ) = 0,\theta (\eta_{0} ) = 0,\varphi (\eta_{0} ) = 0} \\ \end{array} } \right\}$$

where $$\eta_{0} = {\text{tan}}\,\gamma$$.

It is easy to demonstrate the non-dimensional parameters in the problem at hand as follows:

The magnetic parameter is $M = \sigma B_{0}^{2} r^{2} /\rho \upsilon$, Prandtl number is $\Pr = \upsilon (\rho c)_{f} /\alpha$, the Brownian motion parameter is $N_{B} = (\rho c)_{p} D_{B} (C_{w} - C_{\infty } )/(\rho c)_{f} \upsilon$, the heat source/ sink parameter is $Q = Q_{1} \,r^{2} /\rho \,c_{f} \,\upsilon$ , the thermophoretic parameter is $N_{T} = (\rho c)_{p} D_{T} (T_{w} - T_{\infty } )/(\rho c)_{f} \upsilon T_{\infty }$, Schmidt number is $Sc = \upsilon /D_{B}$, the reaction rate parameter is $R_{c} = R_{1} r^{2} /\upsilon$, the local Reynolds number, which is based on the disc angular velocity becomes ${\text{Re}}_{\omega } = \omega \,r^{2} /\upsilon$, the local Reynolds number, which is based on the cone angular velocity develops as ${\text{Re}}_{\Omega } = \Omega \,r^{2} /\upsilon$ , and the Eckert number is $Ec = \upsilon^{2} /r^{2} c_{p} (T_{w} - T_{\infty } )$.

## Organization solutions

The basic goal of any perturbation approach, as known, is to convert the nonlinear partial differential equations into ordinary ones. The HPM^41,42^ will be employed to examine the governing system of nonlinear differential equations given by Eqs. (16–21), with the boundary-conditions (26). In view of the HPM, any differential equation may be separated into linear and nonlinear parts as stated in the introduction Section. The artificial parameter ($q$), which is used to generate what is defined by the Homotopy equation, separates these two parts. Accordingly, the aforementioned equations can be formulated as follows:

27 $$h(F,q) = L_{1} (F) + q\left[ {(1 + \beta^{ - 1} )\left( {\eta^{2} F^{\prime\prime} + 3\eta \,F^{\prime}} \right) + \eta \,F\,F^{\prime} + F^{2} - H\,F^{\prime} + G^{2} - M\,F} \right] = 0$$

28 $$h(G,q) = L_{2} (G) + q\left[ {(1 + \beta^{ - 1} )\left( {\eta^{2} G^{\prime\prime} + 3\eta \,G^{\prime}} \right) + \eta \,F\,G^{\prime} - H\,G^{\prime} - M\,G} \right] = 0$$

29 $$h(H,q) = L_{3} (H) + q\left[ {(1 + \beta^{ - 1} )\left( {\eta^{2} H^{\prime\prime} + 3\eta \,H^{\prime} + H} \right) + \eta \,F\,H^{\prime} - H\,H^{\prime} + H\,F} \right] = 0$$

30 $$\begin{gathered} h(\theta ,q) = L_{4} (\theta ) + q[\eta^{2} \theta ^{\prime\prime} + \eta \,\theta ^{\prime} + \Pr (\eta \,F - H)\,\theta ^{\prime} + \Pr \,N_{B} \,(1 + \eta^{2} )\theta ^{\prime}\varphi ^{\prime} + \hfill \\ \,\,\,\,\,\,\,\,\,\,\,\,\,\,\,\,\,\,\,\,\,\,\,\,\,\,\,\Pr \,N_{T} \,(1 + \eta^{2} )\theta ^{{\prime}{2}} + \Pr \,Ec\,M(F^{2} + G^{2} ) + \Pr \,Q\,\theta ] = 0 \hfill \\ \end{gathered}$$

And

31 $$h(\varphi ,q) = L_{5} (\varphi ) + q\left[ {\eta^{2} \varphi ^{\prime\prime} + \eta \varphi ^{\prime} + Sc(\eta F - H)\varphi ^{\prime} + \frac{{N_{T} }}{{N_{B} }}\left( {(1 + \eta^{2} )\theta ^{\prime\prime} + \eta \theta ^{\prime}} \right) - R_{c} Sc\,\varphi } \right] = 0$$

where $L_{1} \equiv L_{2} \equiv L_{3} \equiv \left( {1 + \beta^{ - 1} } \right)\frac{{d^{2} }}{{d\eta^{2} }}$ and $L_{4} \equiv L_{5} \equiv \frac{{d^{2} }}{{d\eta^{2} }}$ are the linear operators.

The dependent variable can be expanded as follows using the previous technique:

32 $$\chi (\eta ,q) = \chi_{0} (\eta ) + q\chi_{1} (\eta ) + q^{2} \chi_{2} (\eta ) + ...$$

where $\chi (\eta ,q)$ refers to any of the functions $F,\,\,G,\,H,\,\,\theta$ and $\varphi$.

Substituting Eq. (32) into Eqs. (27)–(31), after simplification and comparing the like powers of $q$- terms, one gets:

- **Zero-order system:**
  33 $$\frac{{d^{2} \chi_{0} }}{{d\eta^{2} }} = 0$$

where $\chi_{0}$ stands for $F_{0} ,\,\,G_{0} ,\,\,H_{0} ,\,\,\theta_{0} ,\,{\text{and}}\,\,\,\varphi_{0} ,\,\,\,\,\,$with the appropriate boundary conditions that are given as follows:

34 $$\left. {\begin{array}{*{20}c} {F_{0} (0) = 1,G_{0} (0) = {\text{Re}}_{\omega } ,H_{0} (0) = 0,\,\theta_{0} (0) = 1,\varphi_{0} (0) = 1} \\ {F_{0} (\eta_{0} ) = 0,G_{0} (\eta_{0} ) = {\text{Re}}_{\Omega } ,\,H_{0} (\eta_{0} ) = 0,\theta_{0} (\eta_{0} ) = 0,\varphi_{0} (\eta_{0} ) = 0} \\ \end{array} } \right\}$$

- **First-order system:**
  35 $$\left( {1 + \beta^{ - 1} } \right)\frac{{d^{2} F_{1} }}{{d\eta^{2} }} = M\,F_{0} + H_{0} \,F^{\prime}_{0} + (1 + \beta^{ - 1} )\left( {\eta^{2} F^{\prime\prime}_{0} + 3\eta \,F^{\prime}_{0} } \right) - \eta \,F_{0} \,F^{\prime}_{0} - F_{0}^{2} - G_{0}^{2}$$
  36 $$\left( {1 + \beta^{ - 1} } \right)\frac{{d^{2} G_{1} }}{{d\eta^{2} }} + (1 + \beta^{ - 1} )\left( {\eta^{2} G^{\prime\prime}_{0} + 3\eta \,G^{\prime}_{0} } \right) + \eta \,F_{0} \,G^{\prime}_{0} - H_{0} \,G^{\prime}_{0} - M\,G_{0} = 0$$
  37 $$\left( {1 + \beta^{ - 1} } \right)\frac{{d^{2} H_{1} }}{{d\eta^{2} }} = H_{0} \,H_{0}^{\prime} - (1 + \beta^{ - 1} )\left( {\eta^{2} H^{\prime\prime}_{0} + 3\eta \,H^{\prime\prime}_{0} + H_{0} } \right) - \eta \,F_{0} \,H^{\prime}_{0} - H_{0} \,F_{0}$$
  38 $$\frac{{d^{2} \theta_{1} }}{{d\eta^{2} }} = \Pr (H_{0} - \eta \,F_{0} )\,\theta^{\prime }_{0} - \eta^{2} \theta^{\prime \prime }_{0} - \eta \,\theta^{\prime }_{0} - \Pr \,N_{B} \,(1 + \eta^{2} )\theta^{\prime }_{0} \varphi^{\prime }_{0} - \Pr \,N_{T} \,(1 + \eta^{2} )\theta_{0}^{\prime 2} - \Pr \,Ec\,M(F_{0}^{2} + G_{0}^{2} ) - \Pr \,Q\,\theta_{0}$$

And

39 $$\frac{{d^{2} \varphi_{1} }}{{d\eta^{2} }} = Sc(H_{0} - \eta \,F_{0} )\varphi ^{\prime}_{0} + R_{c} \,Sc\,\varphi_{0} - \eta^{2} \varphi ^{\prime\prime}_{0} - \eta \varphi ^{\prime}_{0} - \frac{{N_{T} }}{{N_{B} }}\left( {(1 + \eta^{2} )\theta ^{\prime\prime}_{0} + \eta \theta ^{\prime}_{0} } \right)$$

with the suitable boundary conditions that are specified as follows:

40 $$\left. {\begin{array}{*{20}c} {F_{1} (0) = 0,G_{1} (0) = 0,H_{1} (0) = 0,\,\theta_{1} (0) = 0,\varphi_{1} (0) = 0} \\ {F_{1} (\eta_{0} ) = 0,G_{1} (\eta_{0} ) = 0,\,H_{1} (\eta_{0} ) = 0,\,\theta_{1} (\eta_{0} ) = 0,\varphi_{1} (\eta_{0} ) = 0} \\ \end{array} } \right\}$$

Finally, the approximate solutions for the non-dimensional velocity components, temperature, and nanoparticles concentration are expressed as follows:

41 $$\chi (\eta ) = \mathop {\lim }\limits_{q \to 1} \left( {\chi_{0} (\eta ) + q\chi_{1} (\eta ) + \cdots } \right)$$

Therefore, the distributions of the functions $F,\,\,G\,,\,H,\,\,\theta$ and $\varphi$ can be listed as follows:

42 $$F(\eta ) = \left( {1 - \frac{\eta }{{\eta_{0} }}} \right) + \frac{{\lambda_{3} }}{12}\left( {\eta^{4} - \eta \eta_{0}^{3} } \right) + \frac{{\lambda_{4} }}{6}(\eta^{3} - \eta \eta_{0}^{2} ) + \frac{{\lambda_{5} }}{2}\left( {\eta^{2} - \eta \eta_{0} } \right)$$

43 $$G(\eta ) = \lambda_{1} \eta + \lambda_{2} + \frac{{\lambda_{1} }}{{12\eta_{0} }}\eta^{4} + \lambda_{1} \left( {\frac{M}{6} - \frac{2}{3}} \right)\eta^{3} + \frac{{M\,\lambda_{2} }}{2}\eta^{2} + \lambda_{6} \eta$$

44 $$H(\eta ) = \frac{{\lambda_{3} }}{15}\eta^{5} + \frac{{\lambda_{4} }}{8}\eta^{4} + \frac{{\lambda_{5} }}{3}\eta^{3} + \frac{{\lambda_{6} }}{2}\eta^{2} + \frac{1}{{2\eta_{0} }}\eta^{2}$$

45 $$\theta (\eta ) = \left( {1 - \frac{\eta }{{\eta_{0} }}} \right) + \frac{{\lambda_{10} }}{12}\eta^{4} + \frac{{\lambda_{11} }}{6}\eta^{3} + \frac{{\lambda_{12} }}{2}\eta^{2} + \lambda_{13} \eta$$

46 $$\varphi (\eta ) = \left( {1 - \frac{\eta }{{\eta_{0} }}} \right) - \frac{Sc}{{12\eta_{0}^{2} }}\eta^{4} + \frac{1}{{6\eta_{0} }}\left( {1 + Sc + \frac{{N_{T} }}{{N_{B} }} - R_{c} \,Sc} \right)\eta^{3} + \frac{{R_{c} }}{2}\eta^{2} + \lambda_{15} \eta$$

The constants $\lambda_{1} ,\lambda_{2} , \ldots ,\lambda_{15}$ will be moved to the "Appendix" to follow the manuscript easily.

The next section seeks to study the effects of various issue parameters on the distributions of nanoparticles**,** velocity, temperature, and concentration. A set of figures will be plotted and discussed in the following Section to confirm these influences.

## Results and discussions

A steady, non-Newtonian nanofluid flow in the cavity between a cone and a disc conforming to Casson model is investigated. As previously seen, the rotating velocities of the cone and disc are taken in consideration. The non-dimensional ordinary differential Eqs. (16)–(21) with the boundary conditions (22) are analyzed by means of the HPM. This discussion is valuable for some applications like cone-plate viscometer or rheometer. In such application**s,** the liquid is placed on a horizontal plate and a shallow cone is placed into it. The angle between the surface of the cone and the plate as well as the rotations of plate and cone play important roles in the mechanism of these instruments. Usually, the plate rotates and the force on the cone is measured^57^. The cone and plate viscometer are a standard test for dynamic viscosity measurements, where non-Newtonian fluids display different viscosities relative to the applied shear rate^44^. This may be important in measuring and controlling viscosities of inks, glues, varnishes…etc. Figure 1b and c represent a graphical and real model of the plate-cone rheometer as a good practical example of the current problem.

**Figure 2:**
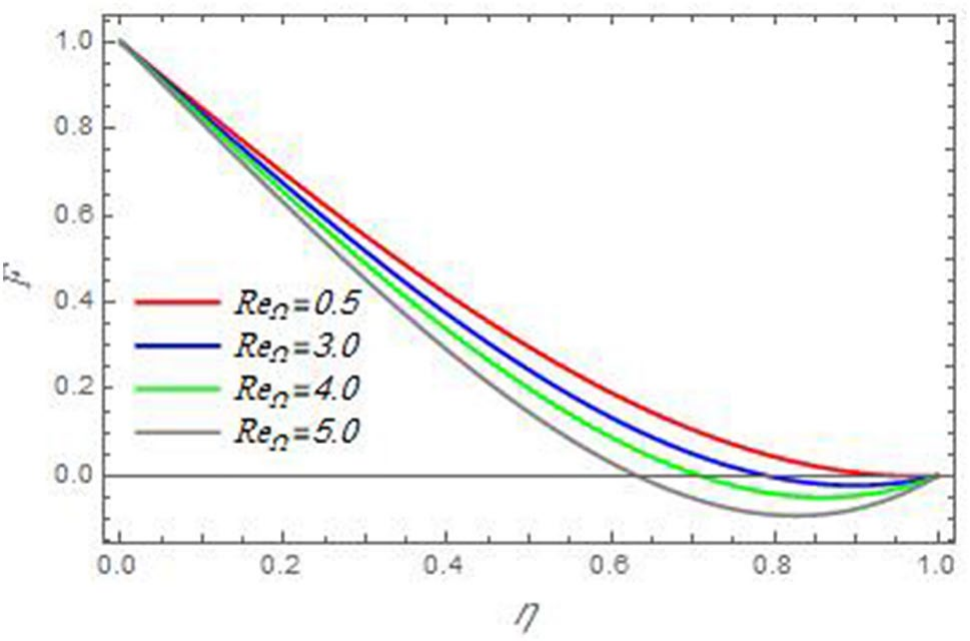
The radial velocity $F$ versus the axial variable $\eta$ in the case of stationary disc to depict the effect of the Reynolds number Re_Ω_.

**Figure 3:**
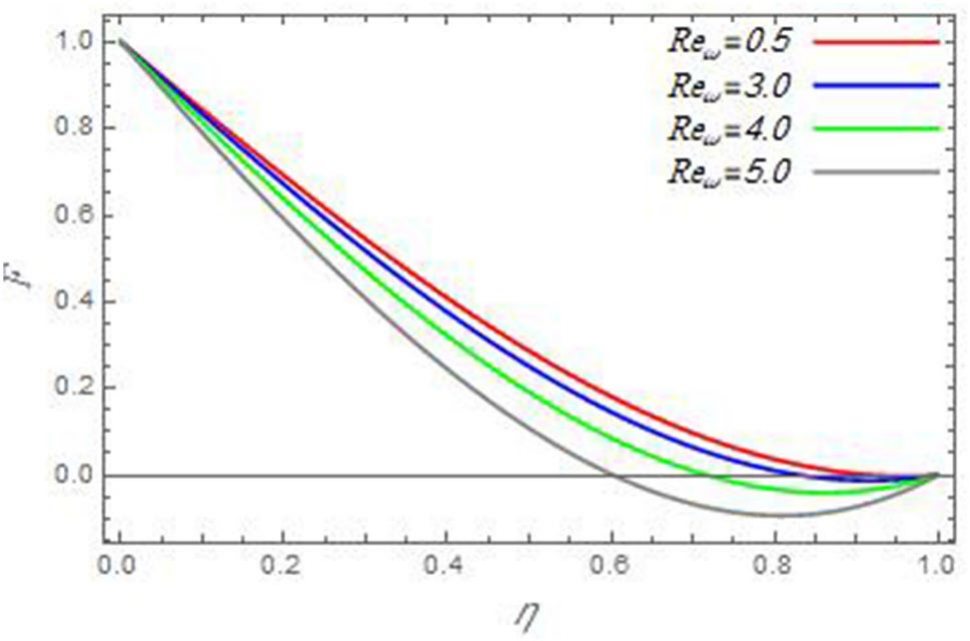
The radial velocity $F$ versus the axial variable $\eta$ in the case of stationary cone to depict the effect of the Reynolds number ${\text{Re}}_{\omega }$.

**Figure 4:**
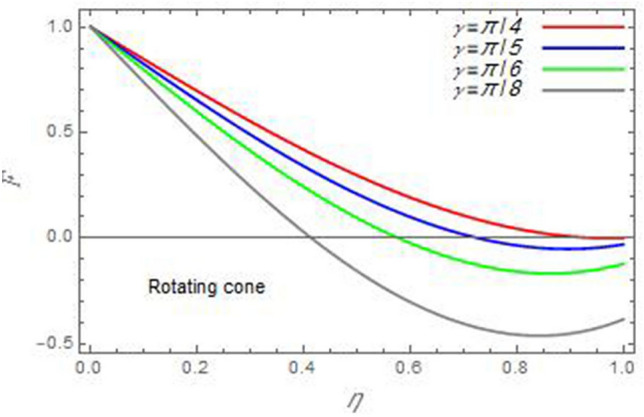
The radial velocity *F* versus the axial variable *η* in the case of stationary disc to depict the effect of the angle between the disc and cone *γ.*

**Figure 5:**
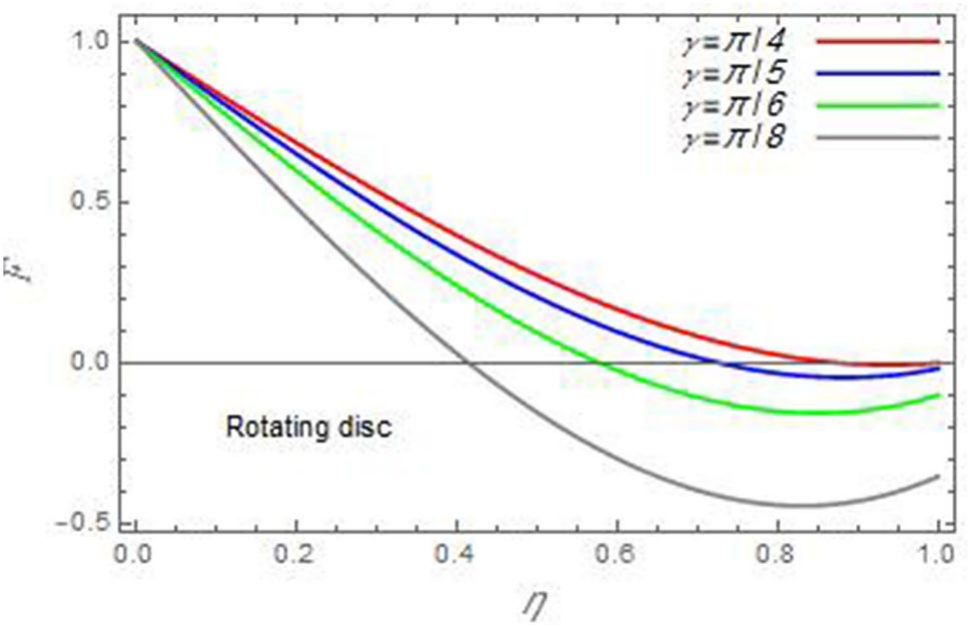
The radial velocity *F* versus the axial variable *η* in the case of stationary cone to depict the effect of the angle between the disc and cone *γ*.

**Figure 6:**
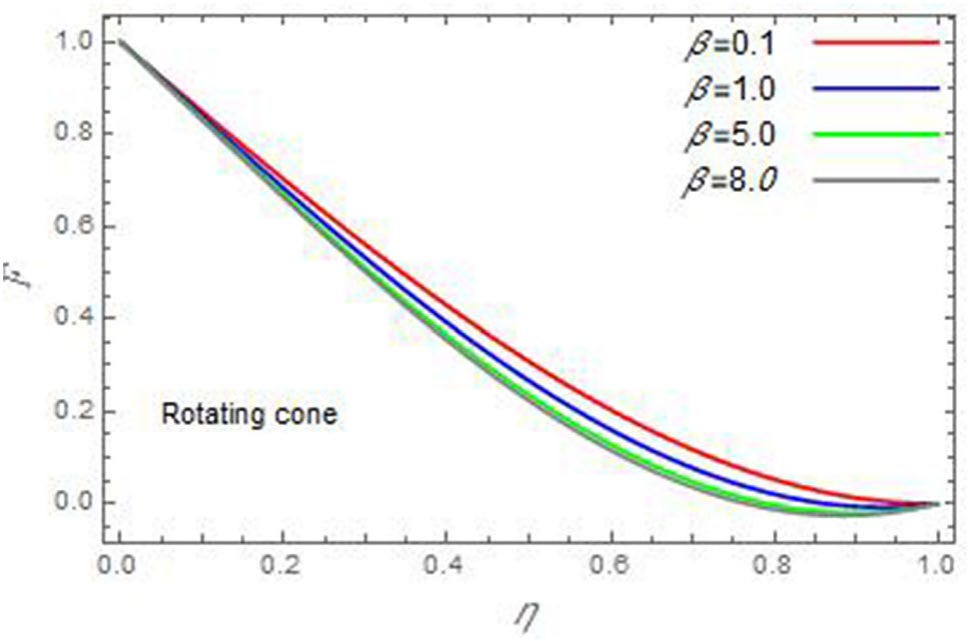
The radial velocity *F* versus the axial variable *η* in the case of stationary disc to depict the effect of the material parameter *β*.

**Figure 7:**
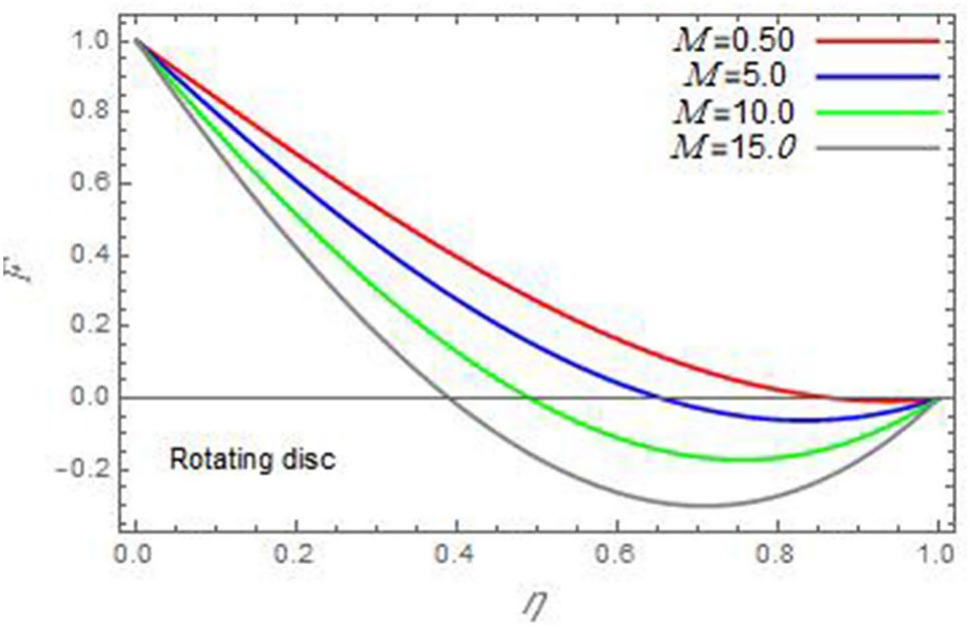
The radial velocity *F* versus the axial variable *η* in the case of stationary cone to depict the effect of the magnetic parameter *M*.

**Figure 8:**
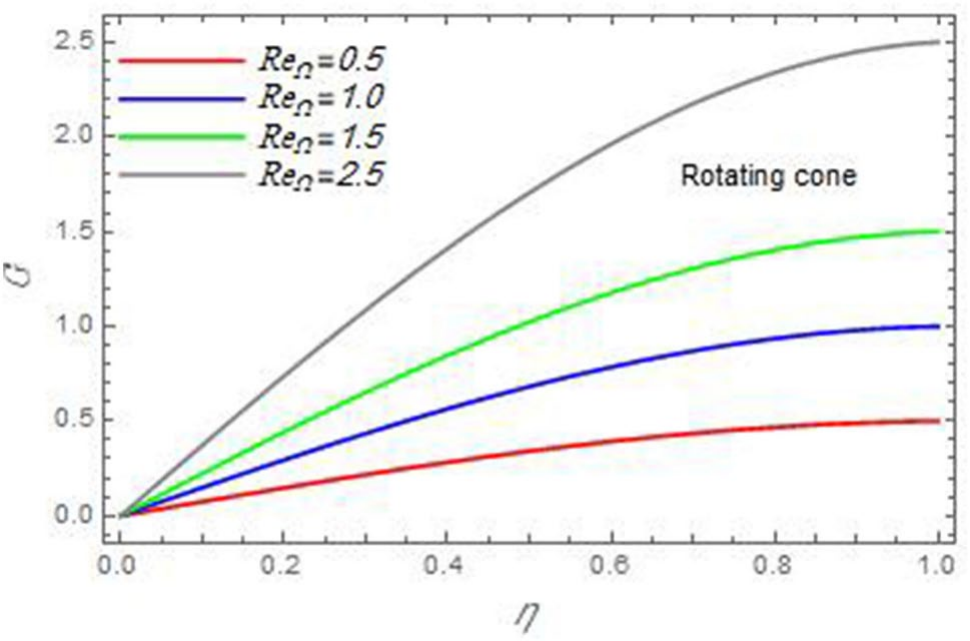
The azimuthal velocity $G$ is plotted versus the axial variable $\eta$ in the case of stationary disc to depict the effect of the Reynolds number ${\text{Re}}_{\Omega }$.

**Figure 9:**
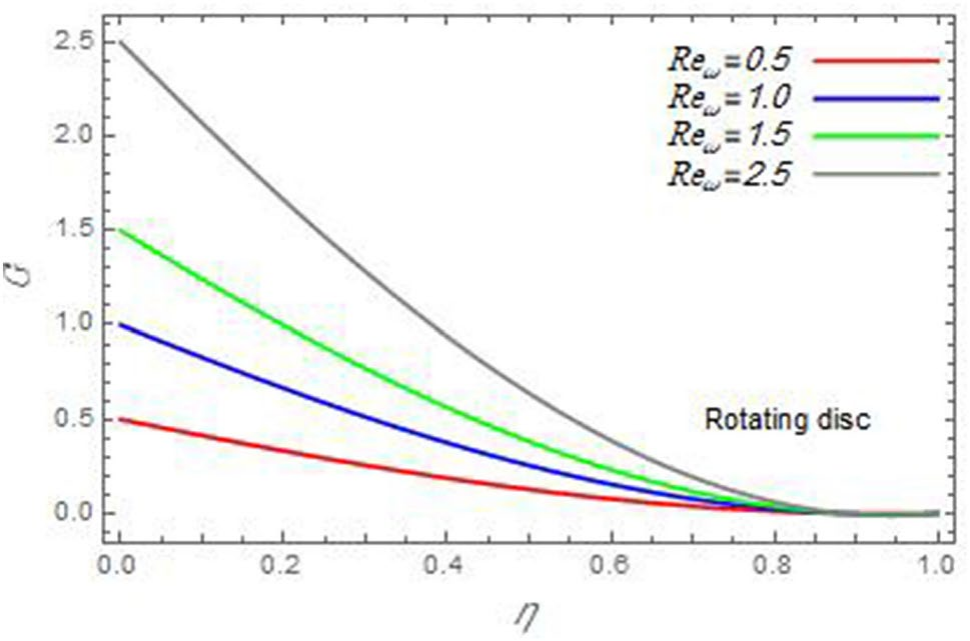
The azimuthal velocity $G$ is plotted versus the axial variable $\eta$ in the case of stationary cone to depict the effect of the Reynolds number ${\text{Re}}_{\omega }$.

**Figure 10:**
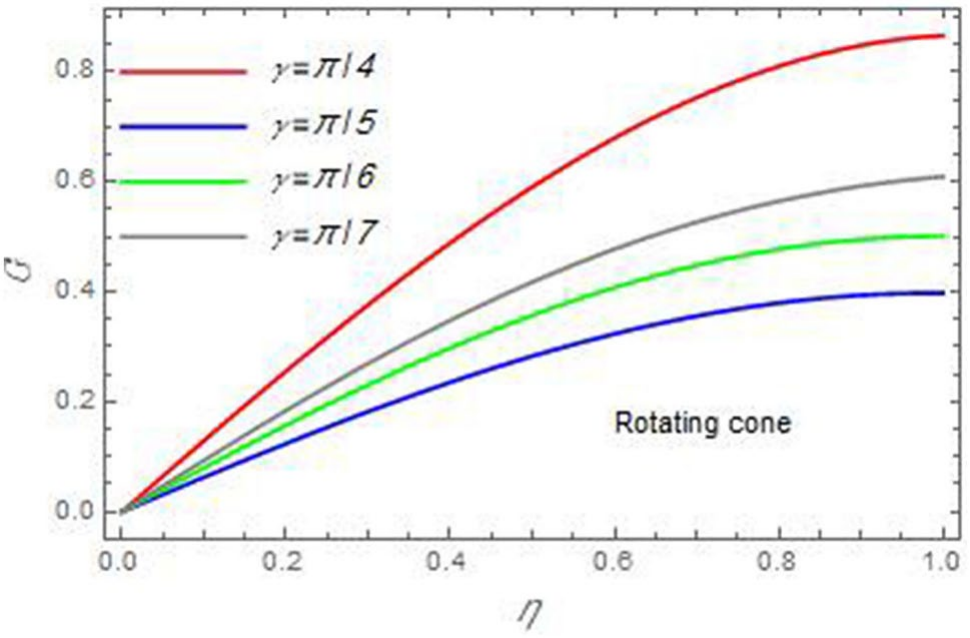
The azimuthal velocity $G$ is plotted versus the axial variable $\eta$ in the case of stationary disc to depict the effect of the angle between the disc and cone $\gamma$.

**Figure 11:**
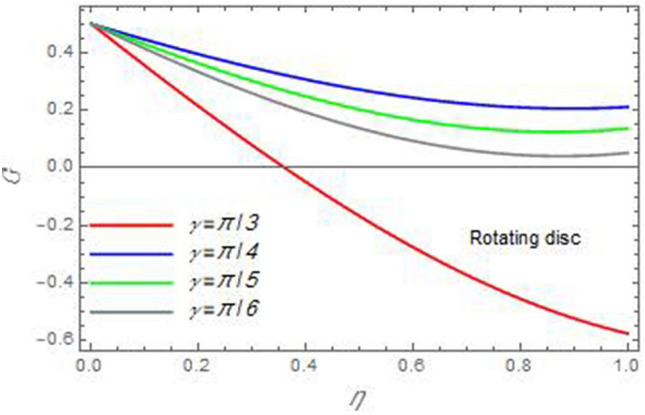
The azimuthal velocity $G$ is plotted versus the axial variable $\eta$ in the case of stationary cone to depict the effect of the angle between the disc and cone $\gamma$.

**Figure 12:**
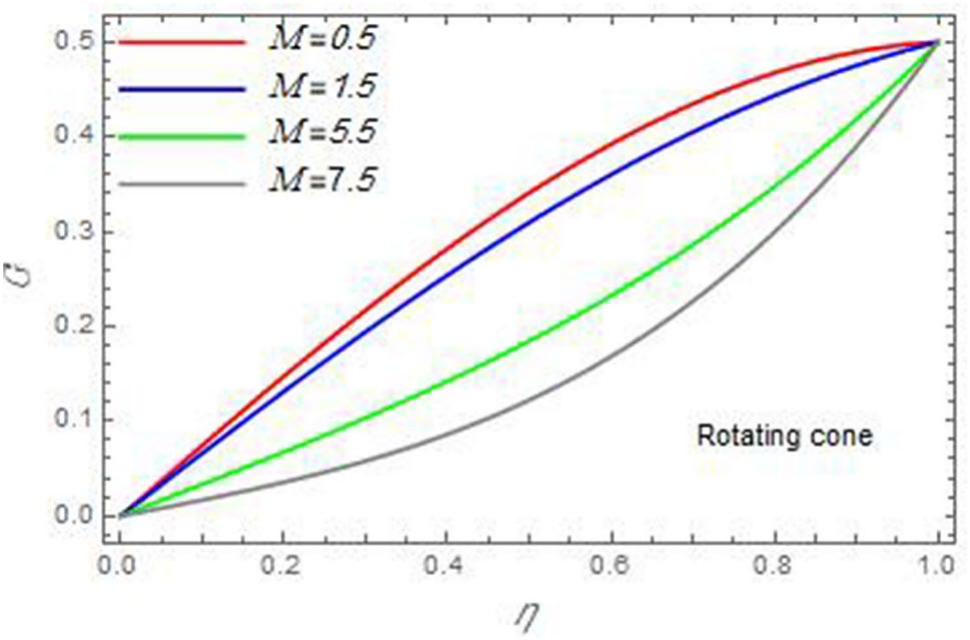
The azimuthal velocity $G$ is plotted versus the axial variable $\eta$ in the case of stationary disc to depict the effect of the magnetic parameter $M$.

**Figure 13:**
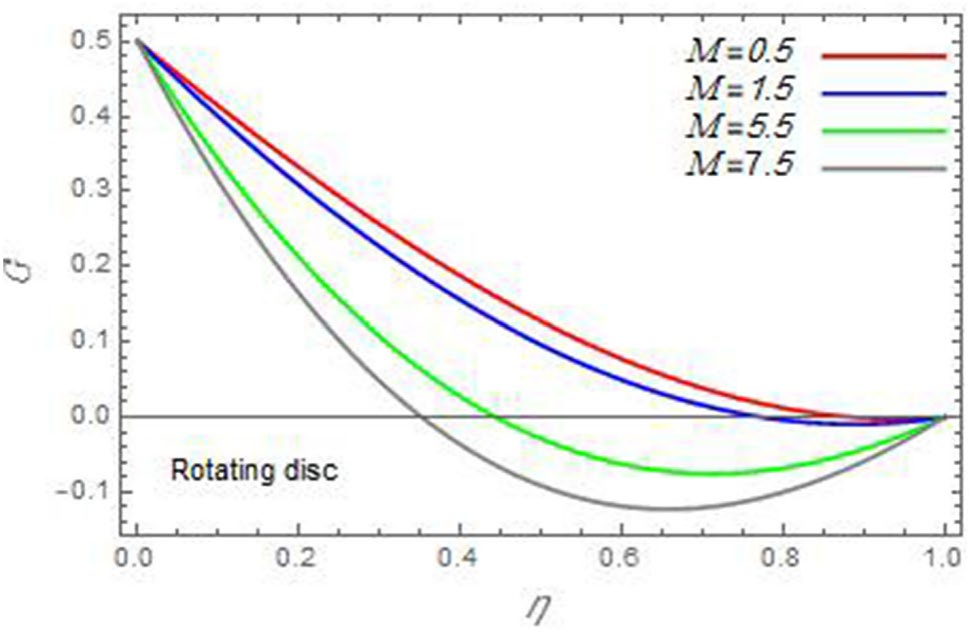
The azimuthal velocity $G$ is plotted versus the axial variable $\eta$ in the case of stationary cone to depict the effect of the magnetic parameter $M$.

**Figure 14:**
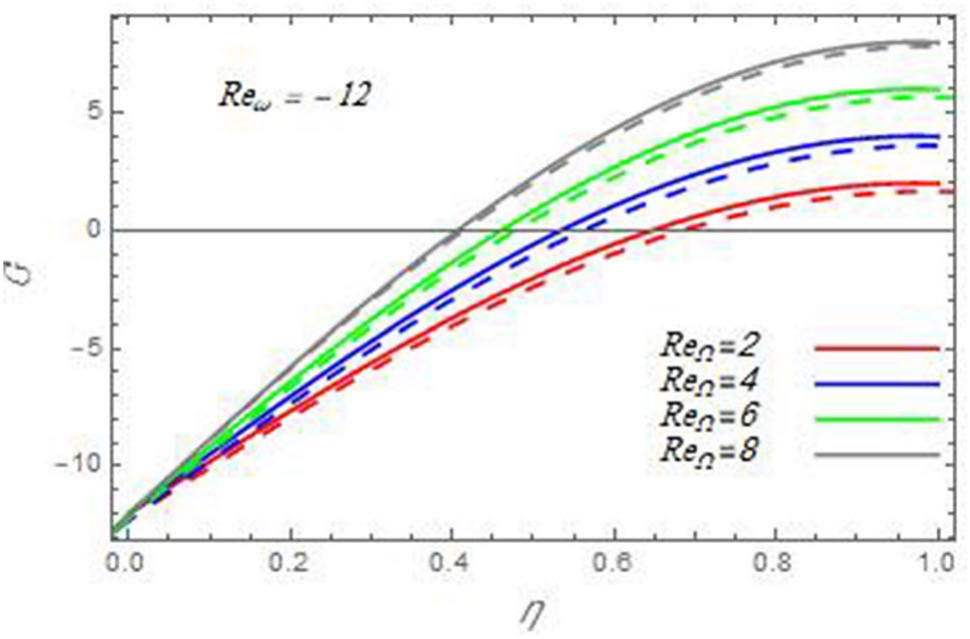
A comparison of the azimuthal velocity $G$ between the current study and Ref. ^58^ in the case ${\text{Re}}_{\omega } = - 12$ with the same values of the Reynolds number ${\text{Re}}_{\Omega }$.

**Figure 15:**
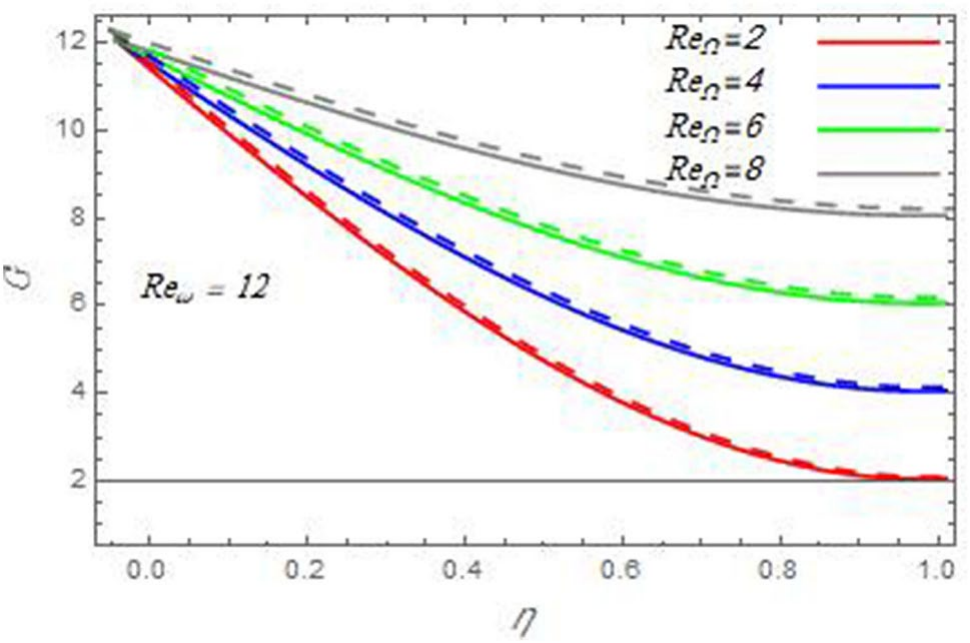
A comparison of the azimuthal velocity $G$ between the current study and Ref. ^58^ in the case ${\text{Re}}_{\omega } = 12$ with the same values of the Reynolds number ${\text{Re}}_{\Omega }$.

**Figure 16:**
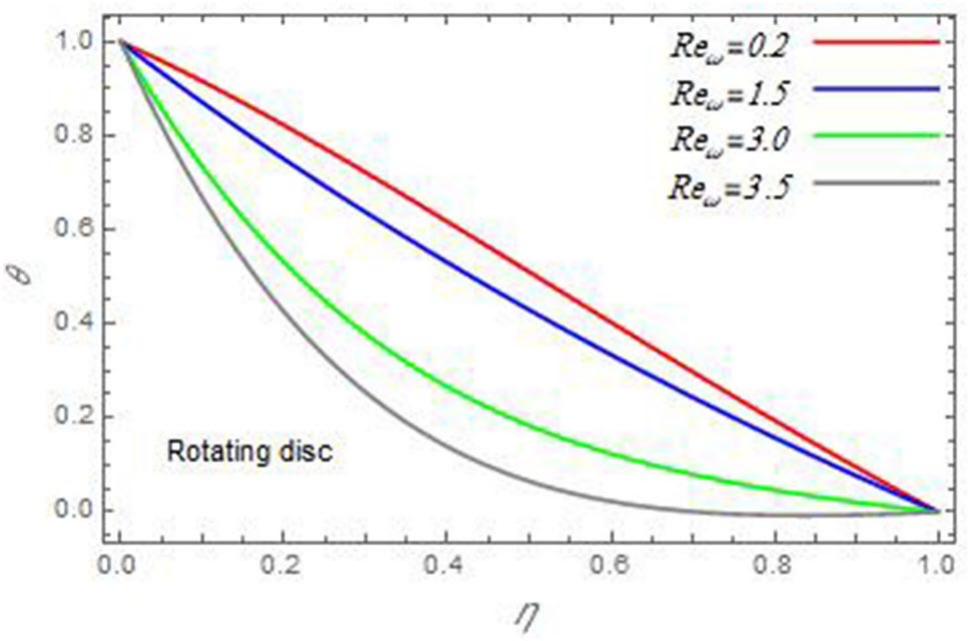
The temperature distribution $\theta$ is plotted versus the axial variable $\eta$ in the case of stationary cone to depict the effect of the Reynolds number ${\text{Re}}_{\omega }$.

**Figure 17:**
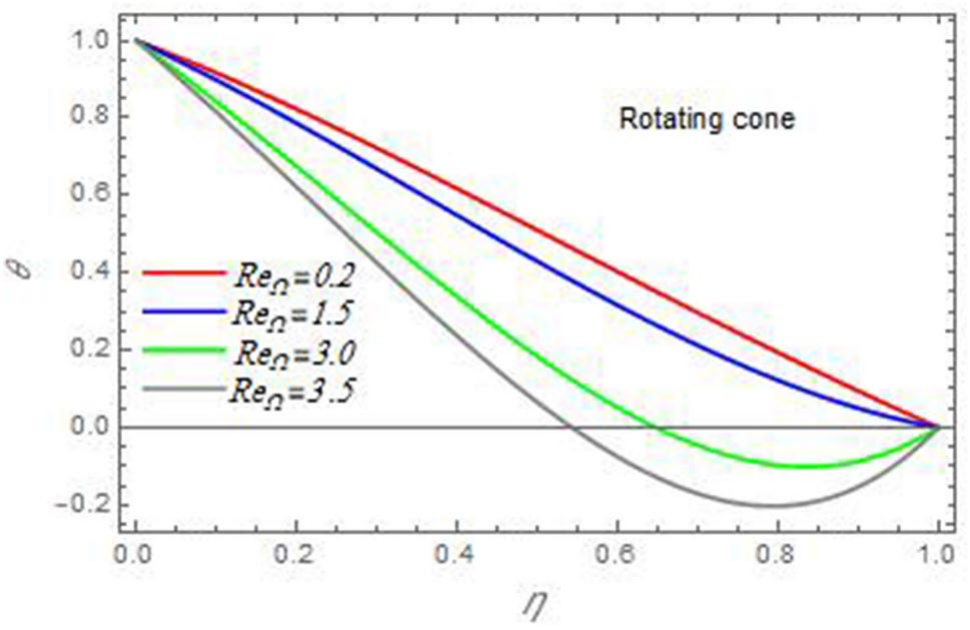
The temperature distribution $\theta$ is plotted versus the axial variable $\eta$ in the case of stationary disc to depict the effect of the Reynolds number ${\text{Re}}_{\Omega }$.

**Figure 18:**
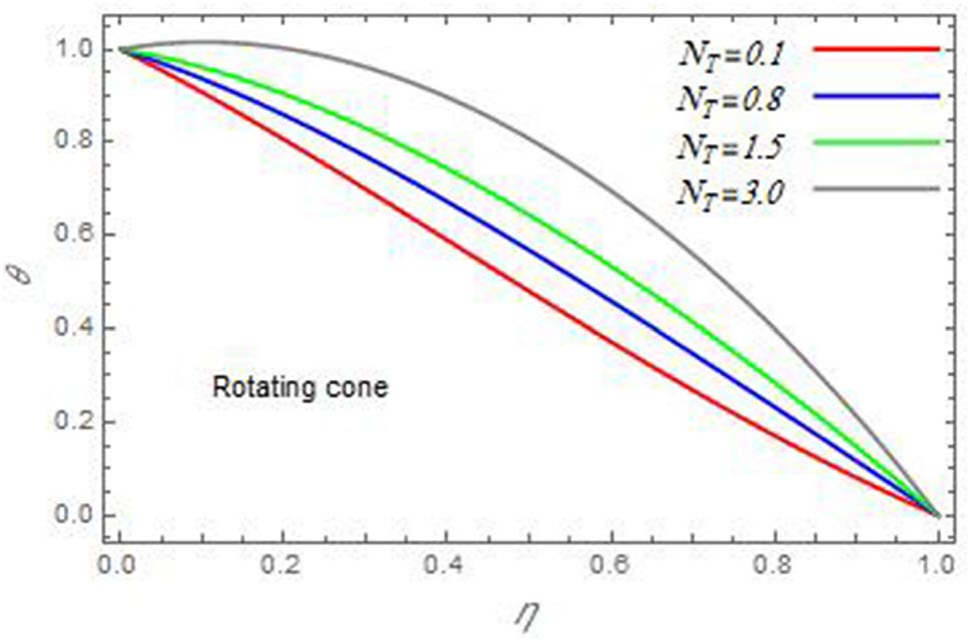
The temperature distribution $\theta$ is plotted versus the axial variable $\eta$ in the case of stationary disc to depict the effect of the thermophoresis parameter $N_{T}$.

**Figure 19:**
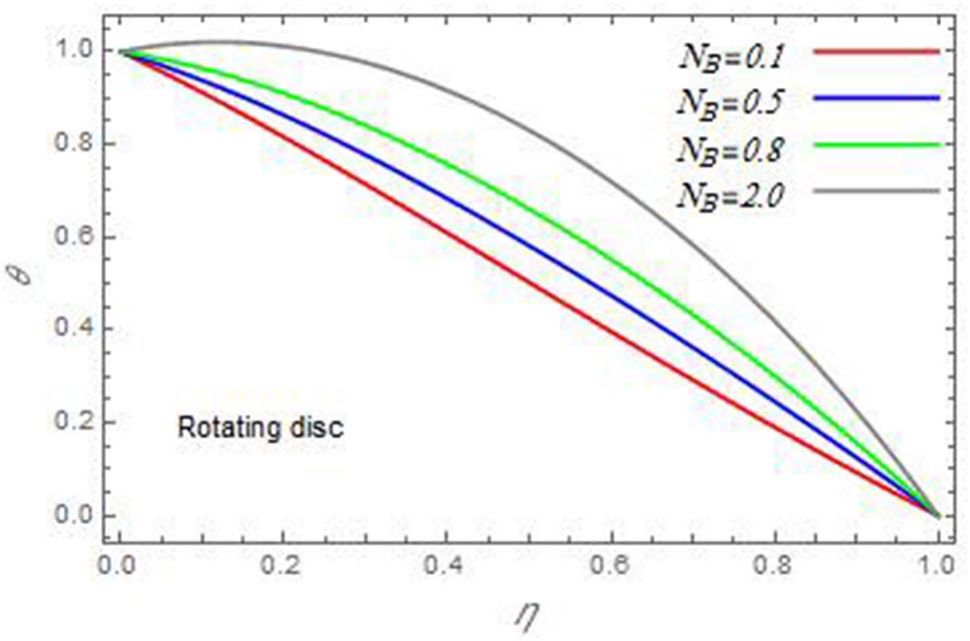
The temperature distribution $\theta$ is plotted versus the axial variable $\eta$ in the case of stationary cone to depict the effect of the Brownian motion parameter $N_{B}$.

**Figure 20:**
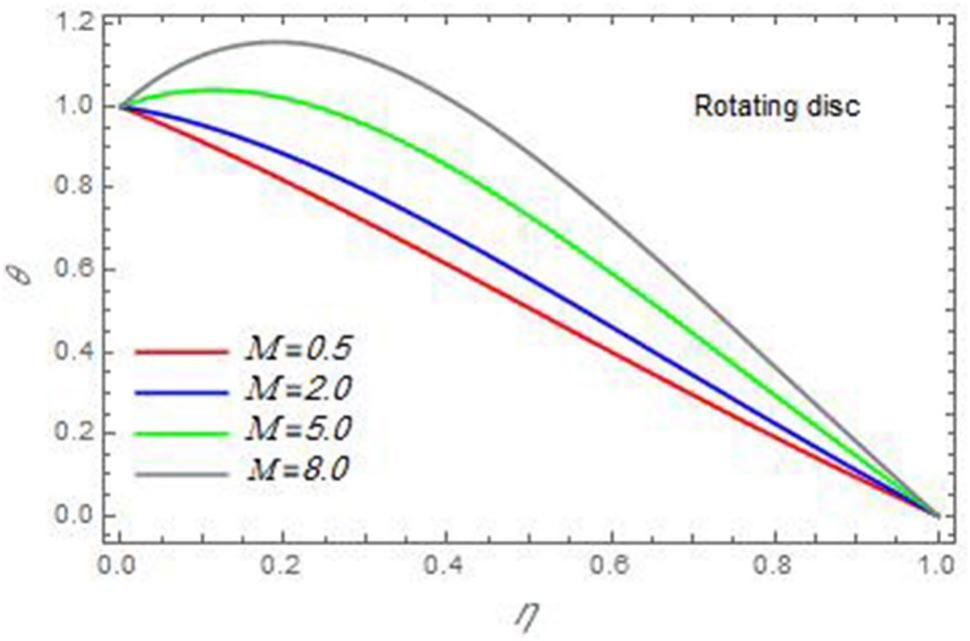
The temperature distribution $\theta$ is plotted versus the axial variable $\eta$ in the case of stationary cone to depict the effect of the magnetic parameter $M$.

**Figure 21:**
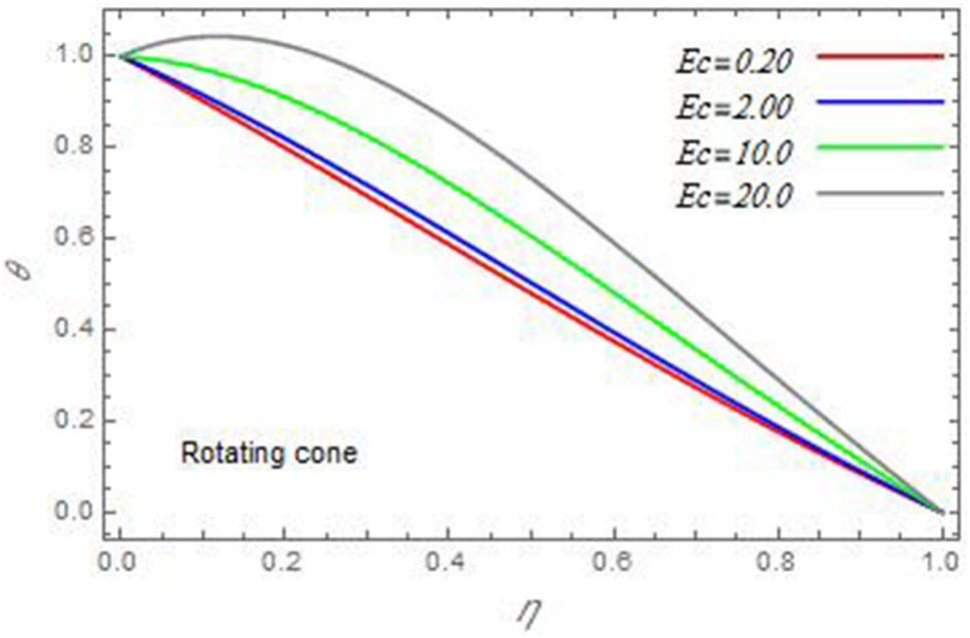
The temperature distribution $\theta$ is plotted versus the axial variable $\eta$ in the case of stationary disc to depict the effect of the Eckert number $Ec$.

**Figure 22:**
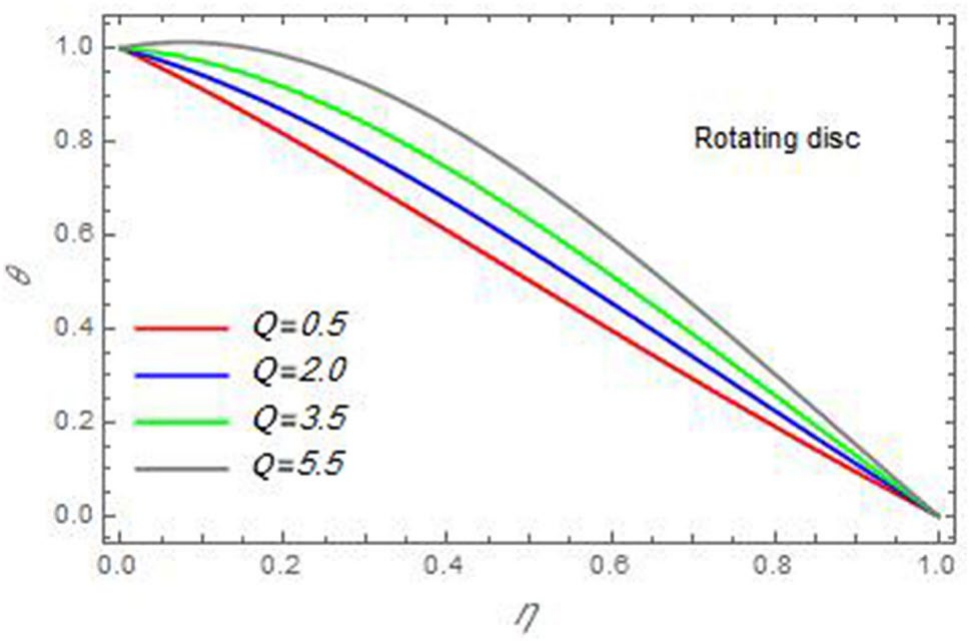
The temperature distribution *θ* is plotted versus the axial variable *η* in the case of stationary cone to depict the effect of the heat source parameter *Q* .

**Figure 23:**
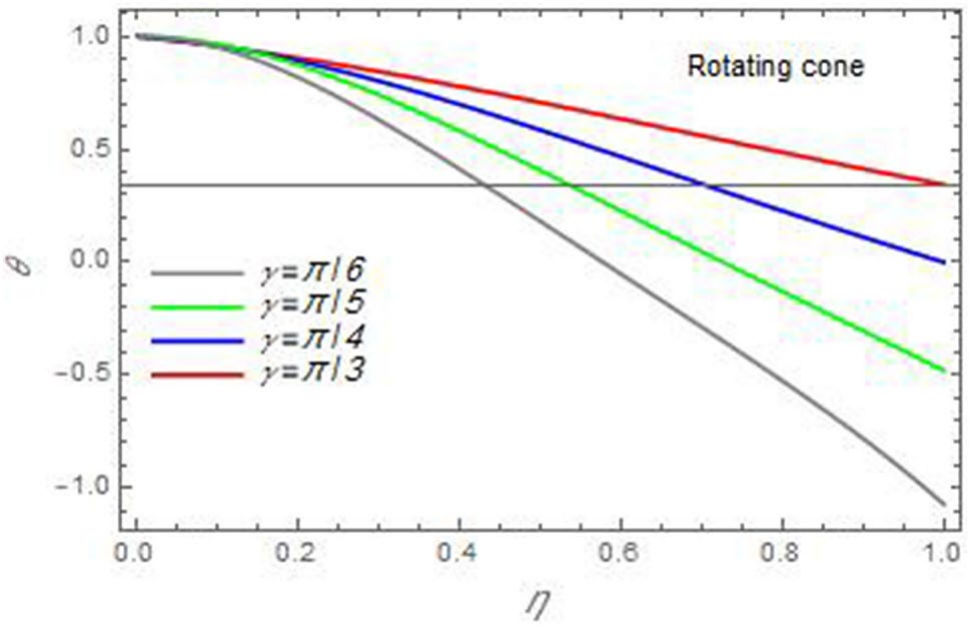
The temperature distribution *θ* is plotted versus the axial variable *η* in the case of stationary disc to depict the effect of the angle between the disc and cone *γ* .

**Figure 24:**
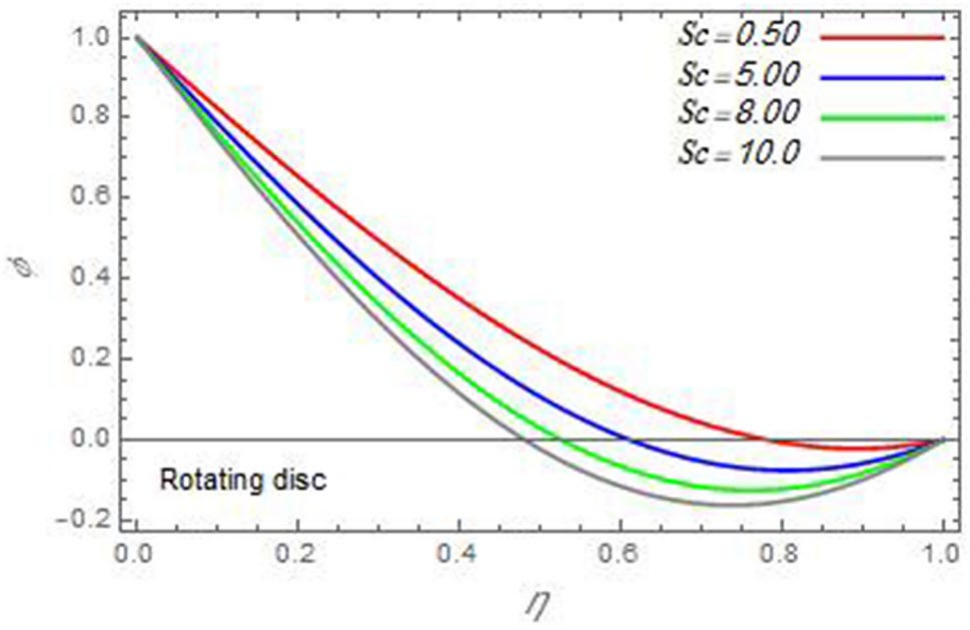
The nanoparticles volume fraction distribution $\varphi$ is plotted versus the axial variable $\eta$ in the case of stationary cone to depict the effect of the Schmidt number $Sc$.

**Figure 25:**
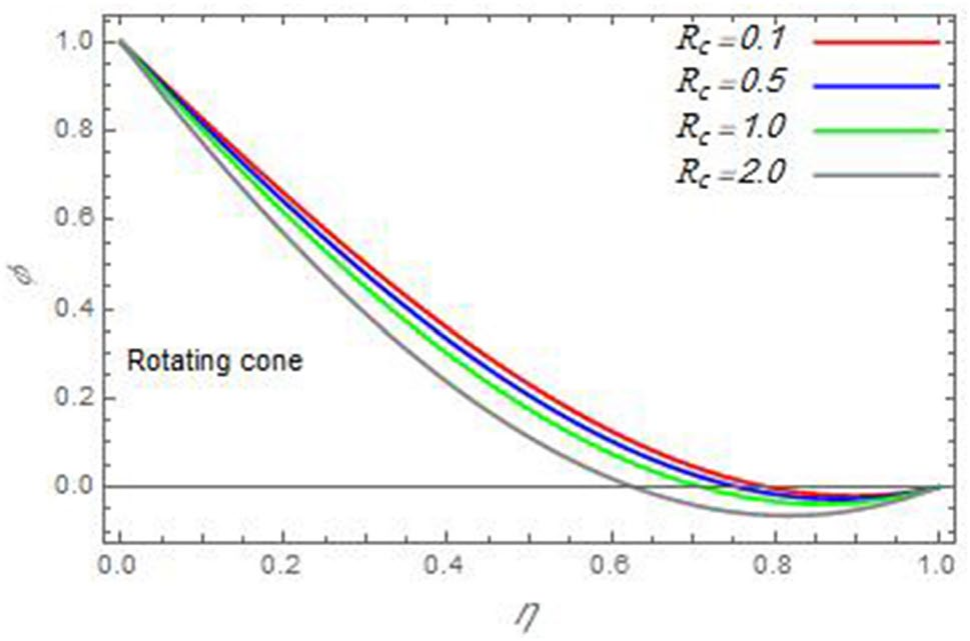
The nanoparticles volume fraction distribution $\varphi$ is plotted versus the axial variable $\eta$ in the case of stationary disc to depict the effect of the chemical reaction parameter $R_{c}$.

In order to clarify the problem physically, the effects of various parameters are discussed, and the results are illustrated through the present Section. The realized parameters include the non-Newtonian fluid parameter $\beta$ the Brownian motion parameter $N_{B}$, the thermophoresis parameter $N_{T}$, the magnetic parameter $M$, the heat source/sink parameter $Q$, the Schmidt number $Sc$, the Prandtl number $\Pr$, the reaction rate parameter $R_{c}$, the local Reynolds number based on the disc angular velocity ${\text{Re}}_{\omega }$, the local Reynolds number based on the cone angular velocity ${\text{Re}}_{\Omega }$ and the Eckert number $Ec$. The following examination focuses on the effects of these parameters on the distributions of velocity, temperature, and nanoparticles distributions. In what follows, the influences of the various physical parameters on the velocity distributions of the radial, axial and azimuthal directions will be displayed throughout Figs. 2, 3, 4, 5, 6, 7, 8, 9, 10, 11, 12, 13, 14, 15, 16, 17, 18, 19, 20, 21, 22, 23, 24 and 25.

In the following figures, for more convenience, the non-dimensional distribution functions are plotted versus the non-dimensional axial variable $\eta$ for some constant values of the parameters, which vary owing to the discussed parameter in each figure, where:

$$\begin{gathered} \Pr = 0.7,\,\,{\text{Re}}_{\Omega } = 0\,or\,0.5,\,{\text{Re}}_{\omega } = 0.5\,or\,0,\,\gamma = \pi /4,\,M = 0.5,\,N_{B} = 0.1,\,N_{T} = 0.3,\,\beta = 0.2,\,Ec = 2,\,R_{c} = 0.2,\,Q = 0.2,\,\, \hfill \\ \,{\text{and}}\,\,\,Sc = 0.5. \hfill \\ \end{gathered}$$

**Figure 26:**
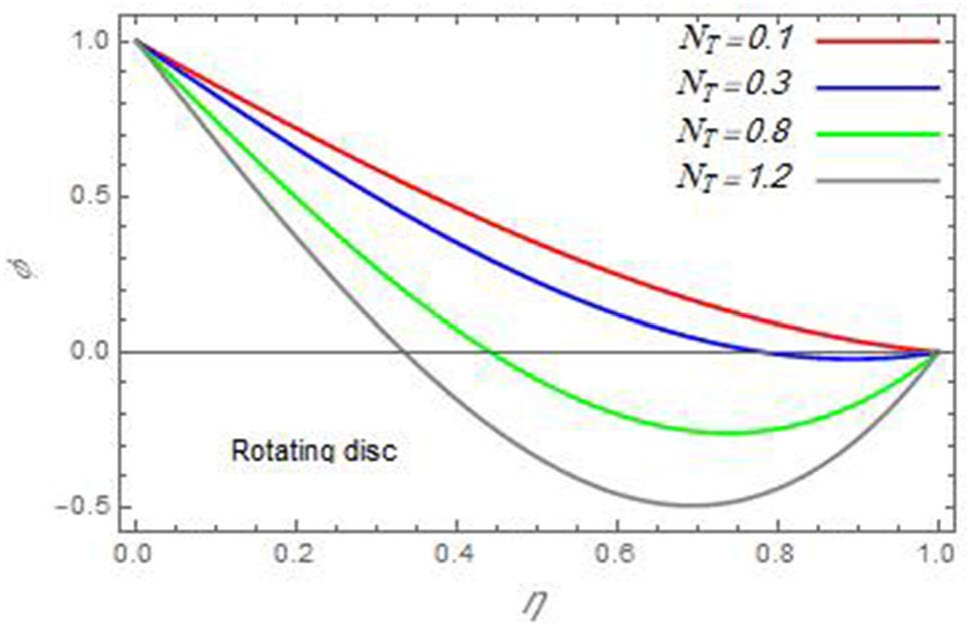
The nanoparticles volume fraction distribution $\varphi$ is plotted versus the axial variable $\eta$ in the case of stationary cone to depict the effect of the thermophoresis parameter $N_{T}$.

**Figure 27:**
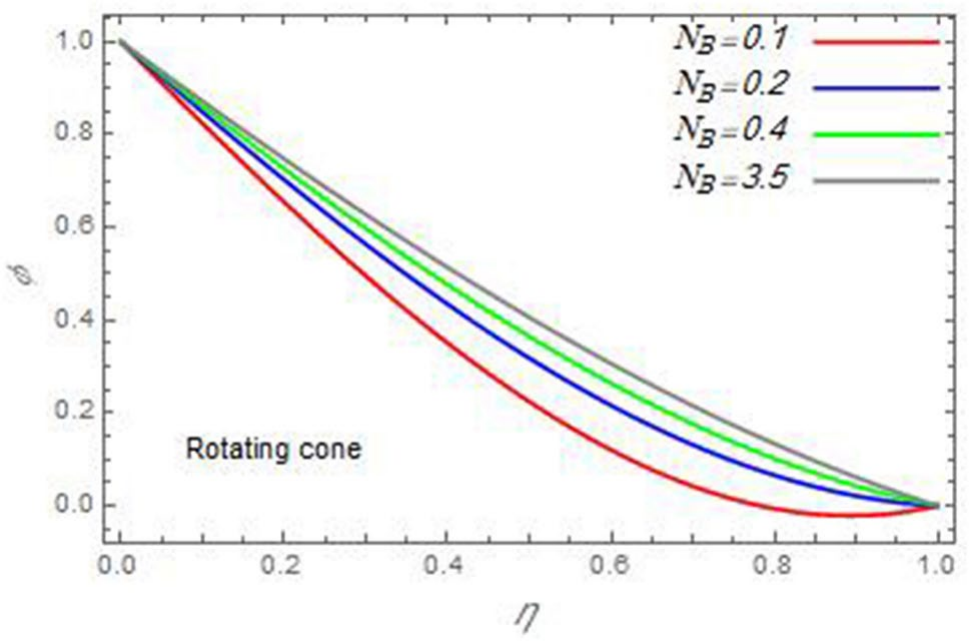
The nanoparticles volume fraction distribution $\varphi$ is plotted versus the axial variable $\eta$ in the case of stationary disc to depict the effect of the Brownian motion parameter $N_{B}$.

- **Velocity distribution**
  In light of the radial velocity, the two cases of the rotating cone/stationary disc and the rotating disc/stationary cone are discussed in Figs. 2, 3, 4, 5 for various relevant parameters. In these figures, the radial velocity $(F)$ is plotted versus the axial variable $\eta$. Therefore, Figs. 2 and 3 are plotted to realize the behavior of the radial velocity with respect to the effects of the two Reynolds numbers ${\text{Re}}_{\Omega }$ and ${\text{Re}}_{\omega }$, respectively. It is found that the radial velocity decreases with the increase of the two Reynolds numbers; meanwhile it vanishes at the free stream. Physically, the Reynolds number represents the ratio between the inertial and viscous forces, which is subjected to relative internal movement of different fluid velocities. This relative movement generates more friction, which leads to a reduction in the radial velocity. This result is compatible with the previous results described by Malik et al.^59^. Figures 4 and 5 indicate that the radial velocity decreases with the increase of the angle $\gamma$. Naturally, the increase of $\gamma$ means an increase in the space between the cone and the disc, which causes a slower motion in the radial direction. Additionally, the influences of the non-Newtonian parameter $\beta$ and the magnetic parameter $M$ of the distribution $F$ are displayed throughout Figs. 6 and 7, respectively. In Fig. 6, the case of the rotating cone/stationary disc is only considered. As seen from this figure, the radial velocity decreases with the increase of the parameter $\beta$. Physically, the parameter $\beta$, as a measure of an extra viscosity of the fluid, reduces the fluid velocity, which agrees with Malik et al.^59^ and Arthur et al.^60^. In Fig. 7, the case of the rotating disc/stationary cone is only reflected. This figure shows the effect of the parameter $M$ on the radial velocity distribution. It is found that the radial velocity decreases with the increase of the parameter $M$. Physically, the Lorentz force obstructs the fluid flow and becomes stronger with the increase of $M$; therefore, the fluid velocity decreases. This result agrees with the results obtained by Mabood et al.^61^, Sabu et al.^62^, Lv et al.^63^ and Alebraheem and Ramzan^64^.
  In view of the distribution of the azimuthal velocity, the two cases of the rotating cone/stationary disc and the rotating disc/stationary cone are illustrated in Figs. 8, 9, 10, 11, 12, 13 for numerous significant parameters. Along with these figures, the azimuthal velocity $(G)$ is graphed versus the axial variable $\eta$. Consequently, Figs. 8 and 9 are designed to understand the behavior of the azimuthal velocity with respect to the effects of the two Reynolds numbers ${\text{Re}}_{\Omega }$ and ${\text{Re}}_{\omega }$, respectively. As seen from these figures, the azimuthal velocity $G$ increases with the increase of both ${\text{Re}}_{\Omega }$ and ${\text{Re}}_{\omega }$ . Owing to the azimuthal velocity acting in the direction of rotation, the increase of ${\text{Re}}_{\Omega }$ and ${\text{Re}}_{\omega }$ naturally leads to an increase in $(G)$. This result corresponds to that reported by Gul et al.^54^. The increase of the angle $\gamma$ has two opposite effects, as seen from Figs. 10 and 11. Figure 10 shows that in the case of the rotating cone/stationary disc, the azimuthal velocity decreases**,** then increases with the increase of $\gamma$. On the other hand, in the case of the rotating disc/stationary cone, Fig. 11 indicates that the azimuthal velocity increases**,** then decreases with the increase of $\gamma$. These results mean that the rotation of the cone as well as the increase of the angle, with the stationary disc, cause a reduction in the azimuthal velocity. This occurs due to the existence of a slip condition at the disc. After that, the effect of $\gamma$ is reflected with its increase due to moving away the disc. Figures 12 and 13 indicate the variation of the azimuthal velocity $G$ with the magnetic parameter $M$. It is found that $G$ decreases with the increase of $M$ in the case of the rotating cone/stationary disc as seen from Fig. 12. The same behavior occurs in the case of the rotating disc/stationary cone, as seen from Fig. 13. The physical interpretation is the same as mentioned above in Fig. 7. These results are compatible with those of Gul et al.^54^ and Nadeem and Saleem^65^. It should be noted that the behavior of the axial velocity distribution versus the axial variable $\eta$, in light of the previous physical parameters, produces similar results as before. Therefore, to restrict the length of the paper, these figures will be excluded. Figures 14 and 15 display a comparison of the azimuthal velocity behavior with the previously published study Wang et al.^58^ with the same values of the two Reynolds numbers ${\text{Re}}_{\Omega }$ and ${\text{Re}}_{\omega }$ and for $M = \beta = 0$. It is seen from these two Figs that the two groups of curves are very closed.
- **Temperature distribution**
  In what follows, the influences of the various physical parameters on the temperature distribution versus the variable parameter $\eta$ are graphed throughout Figs. 16, 1, 18, 19, 20, 21, 22 and 23. Figures 16 and 17 display the effects of the Reynolds numbers ${\text{Re}}_{\Omega }$ and ${\text{Re}}_{\omega }$, respectively, on the $\theta$ profile. As shown from these figures, as ${\text{Re}}_{\Omega }$ and ${\text{Re}}_{\omega }$ increase, the distribution $\theta$ decreases. As previously seen from the physical meaning of ${\text{Re}}_{\Omega }$ and ${\text{Re}}_{\omega }$, the larger values of these parameters lead to a smaller value of viscosity, hence, the temperature decreases. Figures 18 and 19 are designed to indicate the effects of the thermophoresis parameter $N_{T}$ and the Brownian motion parameter $N_{B}$ on heat transfer. It is seen from these two figures that the increase of $N_{T}$ and $N_{B}$ increase the fluid temperature. Physically, the thermophoresis parameter $N_{T}$ increases the movement of nanoparticles from the hot surface to the ambient fluid, which leads to higher temperatures in the surrounding fluid as shown in Fig. 18. Additionally, the Brownian motion parameter $N_{B}$, as a measure of the random motion of the nanoparticles, advances the temperature in the neighborhood layers of fluid as shown in Fig. 19. These findings correspond to Awais et al.^66^, Gbadeyan et al.^67^ and Nadeem et al.^68^. Figures 20 and 21 show the enhancement of the temperature due to the growing of the magnetic parameter $M$ and the Eckert number $Ec$. Physically, as said before, the increase of the magnetic field enhances the Lorentz force that opposes the flow, which in turn enhances the thermal diffusion as seen in Fig. 20. This result is inconsistent with Mabood et al.^61^ and Alebraheem and Ramzan^64^. Moreover, the Eckert number $Ec$ expresses the relationship between the flow kinetic energy and the boundary layer enthalpy difference, and is used to characterize heat transfer dissipation. This heat dissipation produces heat due to contact between the fluid particles, which causes an increase of the original fluid temperature as shown by Fig. 21. This effect of the Eckert number on heat transfer is in accord with that of Abou-Zeid and Mohamed^69^, Eldabe et al.^70^, Anjali Devi and Vasantha Kumari^71^ and Pal and Mandal^72^. The effects of the heat source parameter $Q$ and the angle $\gamma$ on heat transfer are attained by Figs. 22 and 23. It is seen that heat transfer improves with the growth of both of them. Reasonably, the increase of the heat source parameter $Q$ means an increase in the total heat source, which leads to an increase of the heat transfer rate. All research works that involve the heat source like Pal and Mandal^72^, Sabu et al.^62^ and and many other studies, reached the same result. Moreover, **n**aturally, the increases of the angle $\gamma$ means an increase in the area between the cone and the disc which leads to a rise in the temperature distribution. As a conclusion, it is found that most of the physical parameters, involved in this work, improve the heat transfer rate.
- **Nanoparticle volume fraction distribution**
  The final numerical calculations are concerned with the impacts of the various parameters on the nanoparticle volume fraction distribution. Therefore, Figs. 24, 25, 26 and 26 are plotted to display the influences of the parameters: the Schmidt number $Sc$, the chemical reaction parameter $R_{c}$, the thermophoresis parameter $N_{T}$ and the Brownian motion parameter $N_{B}$ of the nanoparticles volume fraction distribution $\varphi$, respectively. As seen from Fig. 24, the nanoparticles distribution $\varphi$ decreases with the increase of the Schmidt number $Sc$. In accordance with the fact that the Schmidt number represents the ratio of momentum to mass diffusivities, the mass diffusivity decreases with the increase of $Sc$, which means a decrease in $\varphi$. This result agrees with that early obtained by Arthur et al.^60^, Mabood et al.^61^ and Sabu et al.^62^. Figure 25 indicates that the nanoparticles concentration decreases with the increase of the chemical reaction parameter $R_{c}$. Physically, as $R_{c}$ increases, an extensive diffusion of mass through the ambient fluid occurs. Hence, it causes scattering nanoparticles away over the flow and leads to a reduction of the nanoparticles concentration. The augmentation in the parameter $N_{T}$ gives a logic and physical explanation to the decrease in $\varphi$, where the nanoparticles disperse and accelerate in their random movement with the increase of $N_{T}$ as shown in Fig. 26. The effect of the Brownian motion parameter $N_{B}$ in the $\varphi$ profile is illustrated in Fig. 27. It is found that the $\varphi$ distribution increases with the increase of $N_{B}$. Physically, Brownian motion is a random motion of particles suspended in a fluid. This random motion increases with the increase of $N_{B}$, which means more divergence of nanoparticles. This result agrees with the findings of Abou-zeid^73^, Abou-zeid and Mohamed^69^ and Alebraheem and Ramzan^64^.
- **Skin friction, Nusselt and Sherwood coefficients**

Tables 1 and 2 are designed to give some results of the local skin friction factors in the radial and tangential directions $Cf_{r}$ and $Cf_{\theta }$, Nusselt $Nu$ and Sherwood $Sh$ numbers for various values of $M$, $\beta$, $\lambda_{1}$ and $Sc$. As seen from Table 1, it is found that $Cf_{r}$, $Cf_{\theta }$ and $Nu$ are reduced by the rise of $M$. Meanwhile, it is found that $Cf_{r}$ increases, $Cf_{\theta }$ decreases and $Nu$ is not affected by the increase of $\beta$. Furthermore, Table 2 shows that the values of $Cf_{r}$, $Cf_{\theta }$ and $Nu$ increase with the rise of $\lambda_{1}$**.** Similarly, the Sherwood number $Sh$ increases with the increase of $Sc$. Some non-variable quantities are excluded from the tables to save space.

**Table 1:** Skin friction along the radial and tangential direction and Nusselt number for some values of the magnetic parameter $M$ and the material parameter $\beta$.

$\beta = 0.2$	$Cf_{r}$	$Cf_{\theta }$	$Nu$	$M = 0.5$	$Cf_{r}$	$Cf_{\theta }$	$Nu$
$M$				$\beta$			
0	− 1.22975	0.659722	1.1175	0	− 1.5	0.75	1.1
0.1	− 1.239	0.652778	1.114	0.1	− 1.37913	0.681818	1.1
0.2	− 1.24826	0.645833	1.1105	0.2	− 1.27604	0.625	1.1
0.3	− 1.25752	0.638889	1.107	0.3	− 1.18713	0.576923	1.1
0.4	− 1.26678	0.631944	1.1035	0.4	− 1.10969	0.535714	1.1
0.5	− 1.27604	0.625	1.1	0.5	− 1.04167	0.5	1.1

**Table 2:** Skin friction along the radial and tangential direction, Nusselt and Sherwood number for some values of the parameter $\lambda_{1}$ , the Schmidt number $Sc$.

$\beta = 0.2$,$M = 0.5$	$Cf_{r}$	$Cf_{\theta }$	$Nu$	$\beta = 0.2$,$M = 0.5$	$Sh$
λ1				$Sc$	
0	− 1.27593	− 0.0416667	1.09917	0	1.28333
0.1	− 1.27512	0.0833333	1.10667	0.1	1.29167
0.2	− 1.27454	0.208333	1.11583	0.2	1.3
0.3	− 1.27419	0.333333	1.12667	0.3	1.30833
0.4	− 1.27407	0.458333	1.13917	0.4	1.31667
0.5	− 1.27419	0.583333	1.15333	0.5	1.325

## Concluding remarks

In accordance with the motivation in many requests in clinical engineering^41^, the current study is done. The theoretical model is concerned with a stationary, non-Newtonian fluid following Casson nanofluid, flowing in a gap between a cone and a disc. These two rigids rotate with different uniform angular velocities or not. For more opportunities, the cylindrical coordinates have been employed. The analysis includes heat transfer with Ohmic dissipation and heat source. Additionally, a nanoparticle volume fraction is calculated under the chemical reaction effect. A uniform magnetic field is pervaded normally to the horizontal rigid disc. The methodology of the problem produced several non-dimensional numbers. For the sake of simplicity, the pressure gradient is ignored. Through suitable similarity transformations, the governing partial differential equations of motion are transformed to another set of nonlinear ordinary ones. Because the HPM is promising and powerful, it is used to analytically solve the nonlinear ordinary equations. Consequently, a set of diagrams has been graphed to illustrate the inclusion of the various physical parameters. When the findings, in the cases of rotating/stationary surfaces of the cone and a horizontal disc, are compared to the previous studies, acceptable influences are drawn. In this study, the effects in the cases of the rotating cone and the rotating disc are similar. Therefore, to avoid redundancy, the figures are delimited by studying only one case interchangeably for some parameters. Generally, based on the theoretical and numerical calculations, the following results are yielded:

- The effect of the magnetic parameter $M$ on all velocity components is qualitatively the same. As shown, the velocity components decrease with the increase of $M$.
- The radial velocity $F$ decreases with the increase of both the two Reynolds numbers ${\text{Re}}_{\Omega }$ and ${\text{Re}}_{\omega }$. Meanwhile, the azimuthal velocity $G$ increases with increase of them.
- Temperature profile increases with the increase of each of the parameters $Q$, $N_{T}$, $N_{B}$, $\gamma$ and $Ec$. Whereas it decreases with the increase of the rotation of cone and disc.
- The nanoparticles distribution $\varphi$ decreases with the increase values of $Sc$, $N_{T}$ and $R_{C}$, meanwhile it increases with the increase of $N_{B}$.
- Some quantitative values of the skin friction factors in the radial and tangential directions $Cf_{F}$ and $Cf_{G}$, Nusselt $Nu$ and Sherwood $Sh$ numbers for various values of $M$, $\beta$, $\lambda_{1}$ and $Sc$ are concluded.

## Supplementary Information

Supplementary Information.

## Data Availability

All data generated or analyzed during this study are included in this manuscript.

## English symbols

*u*,*v*,*w*: Fluid velocity components (m s^−^^1^)
e_ij_: Rate of strain (deformation)
*B*_*o*_: Uniform magnetic field (T(tesla))
*H*: Non-dimensional axial velocity component (m s^-^^−^^1^)
*F*: Non-dimensional radial velocity component (m s^−^^1^)
*G*: Non-dimensional azimuthal velocity component (m s−^1^)
Re_o_: Local Reynolds number based on the disc angular velocity
*M*: Magnetic parameter
*T*: Fluid temperature (K(Kelvin))
*C*: Nanoparticle volume fraction (Mol m^−^^3^)
*D*_*B*_: Brownian diffusivity (m^2^ s^−^^1^)
*D*_*T*_: Thermophoretic diffusion (m^2^ s^−^^1^)
$T_{\infty }$: Temperature of the ambient fluid at the cone (K(Kelvin))
$C_{\infty }$: Nanoparticle volume fraction at the cone (Mol m^−^^3^)
$T_{w}$: Fluid temperature at the disc (K(Kelvin))
$C_{w}$: Nanoparticle volume fraction at the disc (Mol m^−^^3^)
$Q_{1}$: Heat source/sink parameter (m^3^ s^−^^1^)
${\text{Re}}_{\Omega }$: Local Reynolds number based on the cone angular velocity
$R_{c}$: Reaction rate constant
$J_{i}$: Electric current density components
$$N_{T}$$: Thermophoretic parameter
$N_{B}$: Brownian motion parameter

## Greek symbols

$$\rho$$: Density (kg m^−^^3^)
$\tau_{ij}$: Cauchy stress components
$$\sigma$$: Electrical conductivity (s m^−^^1^)
$\mu_{0}$: Plastic dynamic viscosity (kg m^−^^1^ s^−^^1^)
$\omega$: Disc angular velocity (Rad s^−^^1^)
$\Omega$: Cone angular velocity (kg m^−^^1^ s^−^^1^)
$$\upsilon$$: Kinematic viscosity (m^2^ s^−^^1^)
$\beta$: Coefficient of non-Newtonian Casson fluid
$(\rho c)_{f}$: Heat capacity of the fluid (J (Joule))
$(\rho c)_{p}$: Heat capacity of the nanoparticles (J (Joule))
$\eta$: Non-dimensional similarity coordinate
$\theta$: Non-dimensional temperature
$\varphi$: Non-dimensional nanoparticle volume fraction (mol m^−^^3^)
$$\alpha$$: Thermal conductivity (W/(m⋅K))
$\pi$: Product of the component of deformation rate with it
$\pi_{c}$: Critical value based on the non-Newtonian model
$\gamma$: Gap angle between the disc and cone (rad)
