# Supplementary material for: A Casson nanofluid flow within the conical gap between rotating surfaces of a cone and a horizontal disc
Source: Sci Rep. 2022 Jul 4;12:11275. doi: 10.1038/s41598-022-15094-w (PMC9253054; doi:10.1038/s41598-022-15094-w)
Supplement: Supplementary file 1 — Supplementary Information. [file 41598_2022_15094_MOESM1_ESM.docx]

**Appendix**

The constants that appearing in Eqs. (38-42) may be listed as follows:

$\lambda_{1}=\frac{\mathrm{Re}_{\Omega}-\mathrm{Re}_{\omega}}{\eta_{0}}$, $\lambda_{2}=\mathrm{Re}_{\omega}$, $\lambda_{3}=\frac{1}{1+\beta^{-1}}\left(\lambda_{1}^{2}-\frac{2}{\eta_{0}^{2}}\right)$, $\lambda_{4}=\frac{3}{\eta_{0}}+\frac{1}{1+\beta^{-1}}\left(\frac{3+M}{\eta_{0}}-2\lambda_{1}\lambda_{2}\right)$,

$\lambda_{5}=\frac{1}{1+\beta^{-1}}\left(M+\lambda_{2}^{2}-1\right)$,$\lambda_{6}=-\frac{\lambda_{3}}{12}\eta_{0}^{3}-\frac{\lambda_{4}}{6}\eta_{0}^{2}-\frac{\lambda_{5}}{2}\eta_{0}$, $\lambda_{7}=0$, $\lambda_{8}=\lambda_{1}\eta_{0}^{2}\frac{7}{12}-\frac{M}{6}-\frac{1}{2}M\lambda_{2}\eta_{0}$, $\lambda_{9}=0$,

$\lambda_{10}=\lambda_{1}^{2}-\frac{\Pr}{\eta_{0}^{2}}\left(1+N_{B}+N_{T}+Ec M\right)$,$\lambda_{11}=2\lambda_{1}\lambda_{2}+\frac{1}{\eta_{0}}\left[1+\Pr\left(1+2Ec M+Q\right)\right]$,$\lambda_{12}=\lambda_{2}^{2}-\frac{\Pr}{\eta_{0}^{2}}\left(N_{B}+N_{T}+Ec M\eta_{0}^{2}+Q\eta_{0}^{2}\right)$, $\lambda_{13}=-\frac{\lambda_{10}}{12}\eta_{0}^{3}-\frac{\lambda_{11}}{6}\eta_{0}^{2}-\frac{\lambda_{12}}{2}\eta_{0}$,

$\lambda_{14}=0$ and $\lambda_{15}=\left[\frac{Sc}{12}-\frac{1}{6}\left(1+Sc+\frac{N_{T}}{N_{B}}-R_{c}\right)-\frac{R_{c}}{2}\right]\eta_{0}$.
